# The Integrated Study on the Chemical Profiling to Explore the Constituents and Mechanism of Traditional Chinese Medicine Preparation Huatuo Jiuxin Pills Based on UPLC-Q-TOF/MS^E^ and Network Pharmacology

**DOI:** 10.3389/fmolb.2022.818285

**Published:** 2022-03-31

**Authors:** Yulong Zhu, Yaqin Zhu, Shuyue Tao, Wanhui Liang, Jing Zhang, Yunjing Zhang, Zihua Xuan, Jingjing Xu, Can Peng, Huan Wu, Deling Wu

**Affiliations:** ^1^ School of Pharmacy, Anhui University of Chinese Medicine, Hefei, China; ^2^ Anhui Province Key Laboratory of Chinese Medicinal Formula, Hefei, China; ^3^ Anhui Province Key Laboratory of Pharmaceutical Preparation Technology and Application, Hefei, China; ^4^ Synergetic Innovation Center of Anhui Authentic Chinese Medicine Quality Improvement, Hefei, China

**Keywords:** huatuo jiuxin pills, UPLC-Q-TOF/MSE, Cardiovascular Diseases, network pharmacology, molecular docking

## Abstract

Huatuo Jiuxin Pills (HJP), a traditional Chinese medicine (TCM) preparation, has been widely used to treat Cardiovascular Diseases (CVDs) for more than 20 years. However, there were still gaps in the study of chemical components and potential pharmacological effects in the HJP. In this study, ultra-performance liquid chromatography-quadrupole time-of-flight mass spectrometry (UPLC-Q-TOF/MSE) combined with network pharmacology was used to comprehensively explore the chemical components in HJP and explore its potential active compounds and the mechanism for the treatment of CVDs. A total of 117 compounds, mainly including saponins, cholic acids, and bufadienolides, were rapidly identified and characterized. Simultaneously, the fragmentation mode and characteristic ion analysis of different types of representative compounds were carried out. Network pharmacology results showed that the more important active ingredients mainly include 5β‐hydroxybufotalin, 19 oxo‐cinobufagin, bufarenogin, etc. While, the main targets were PIK3CA, MAPK1, VEGFA and so on. Importantly, HJP has therapeutic effects on CVDs by acting on endocrine resistance, PI3K-Akt signaling pathway, HIF-1 signaling pathway, etc. In addition, molecular docking results showed that the core active ingredients with higher degrees in HJP have a strong affinity with the core targets of CVDs. The current work fills the gap in the chemical substance basis of HJP, and also facilitates a better understanding of the effective components, therapeutic targets, and signaling pathways of HJP in the treatment of CVDs.

## 1 Introduction

Cardiovascular Diseases (CVDs) were diseases with high morbidity and mortality (Taskinen and Borén, 2015; Halaris, 2017). Many risk factors may cause CVDs individually or in combination (Deprince et al., 2020). Currently, pharmaceutical chemicals are mainly used in clinical treatment with rapid and direct curative effects. They were usually used with other medicines, but long-term use was prone to adverse reactions (Damiani et al., 2020). Compared with chemical medications, traditional Chinese medicine (TCM) has the characteristics of multiple components, multiple targets, and fewer adverse effects (Wang et al., 2021a). So, it was more in line with the features of long-term medication for the treatment of CVDs. However, due to the complexity of components in TCM preparations, it is still a challenge to identify its effective ingredients, elucidate the mechanism, and discover the relationship between ingredients and therapeutic objectives (Luo et al., 2013).

Huatuo Jiuxin Pills (HJP), a classical TCM preparation, has been clinically used to treat CVDs in China for more than 20 years. It comprises eight crude TCM, i.e., *Panax ginseng C. A. Meyer* (Ren-Shen), *Panax notoginseng (Burk.) F. H. Chen* (San-Qi), *Venenum Bufonis* (Chan-Su), *Borneolum* (Bing-Pian), Artificial Calculus Bovis (Ren-Gong-Niu-Huang), Artificial Moschus (Ren-Gong-She-Xiang), Ox Bile Powder (Niu-Dan-Fen) and Pearl (Zhen-Zhu). The clinical study found that HJP has the function of promoting blood circulation, removing blood stasis, resolving phlegm and dredging collaterals (Zhou et al., 2020). In addition, Zhou’s (Zhou et al., 2020) research also showed that HJP can significantly improve the blood stasis syndrome of experimental myocardial ischemia rats, and reduce its LDH and CPK levels in serum. However, the effective ingredients and *in vivo* action targets of HJP are still unknown, which limits the systematic understanding of the mechanism of HJP action.

In recent years, ultra-performance liquid chromatography-quadrupole time-of-flight mass spectrometry (UPLC-Q-TOF/MS^E^) technology has provided a powerful method for efficient separation and structural characterization of TCM with high resolution, sensitivity, and accuracy (Xu et al., 2020). It is possible to obtain an accurate component precursor ion mass and fragment ions in the full scan mode, while increasing the reliability of the analysis results. And the UNIFI software was a multi-functional automatic data processing platform that can help process mass spectrometry data and quickly analyze chemical components. On the other hand, the mechanism of HJP in the treatment of CVDs and the key active compounds is still unclear. Faced with the current situation, we need an effective means to explore the relationship and mechanism between active components in HJP and core targets. The TCM network pharmacology method combined computational and experimental methods to analyze preparations and their action targets (Wang et al., 2021b), to understand the mechanism of action based on the biological molecular networks of diseases and syndromes. Furthermore, this method can also determine the network regulation mechanism and biological function of TCM, and discover new active compounds. It uncovers “drug-gene-disease” correlations, which are predictive and quantitative measures of the mechanism of action of TCM preparation in disease treatment (Li and Zhang, 2013).

The present study adopted the integration strategy of UPLC-Q-TOF/MS^E^ combined with the UNIFI platform to reveal the effective ingredients of HJP systematically. At the same time, network pharmacology and molecular docking were used to explore the active compounds of HJP and predict its potential targets and signal pathways for CVDs treatment. This study provides a certain basis and reference for the further study of the pharmacodynamic material basis and *in vivo* mechanism of HJP.

## 2 Materials and Methods

### 2.1 Reagents and Materials

HJP was provided by Anhui Bozhou Huatuo National Pharmaceutical Co., Ltd. (Lot number: 20200601). Reference substances including ginsenoside Rd, pseudoginsenoside D, notoginsenoside R_1_, ginsenoside Re, cinobufagin, bufalin, bufotalin, resibufogenin and cholic acid were all purchased from Chengdu Kromah Biotechnology Co., Ltd. Methanol and acetonitrile (MS grade) were purchased from Sigma-Aldrich (Shanghai) Trading Co., Ltd. Ultrapure water was obtained with the PALL laboratory water purification system (PALL, United States).

### 2.2 Standards and Sample Preparation

HJP was ground into powder and 0.3 g of powder was accurately weighed, dispersed in 30 ml methanol, extracted with ultrasonic (360 W, 40 kHz) in the water bath for 30 min. And then, it was filtrated through 0.22 μm filter membrane and the subsequent filtrate was transferred into autosampler vials for UPLC-Q-TOF/MS^E^ analysis. Nine reference standards (ginsenoside Rd, pseudoginsenoside D, notoginsenoside R_1_, ginsenoside Re, cinobufagin, bufalin, bufotalin, resibufogenin and cholic acid) were dissolved in methanol and filtered through 0.22 μm filter membrane to prepare 1 mg/ml stock solution, respectively.

### 2.3 Chromatography and Mass Spectrometry Conditions

Chromatographic analysis was performed used Waters ACQUITY™ UPLC system (Waters Company, Milford, United States). Chromatographic separation was carried out at 30°C, using East Laboratory Epic C_18_ column (100 mm × 2.1 mm, 1.8 μm) with mobile phases A (water) and B (acetonitrile).

The gradient profile was as follows: 0–4 min, 10–15% B; 4–5 min, 15–30% B; 5–11 min, 30% B; 11–15 min, 30–45% B; 15–21 min, 45–80% B; 21–29 min, 80–70% B; 29–30 min, 70–10% B. The flow rate was 0.2 ml/min, and the injection volume was 2 μL.

Mass spectrometric detection was performed on a Waters Xevo G2 Q-TOF mass spectrometer (Waters Corporation, Milford, United States) equipped with an ESI source. The full scan data was acquired from 50 to 1,200 Da, acquisition rate of 0.1 s, source temperature of 100°C, the source voltage of 3.2 kV. The collision voltage was set to 6.0 eV for low-energy scanning and 20–60 eV for high-energy scanning. The nitrogen (N2) temperature was 300°C. In order to obtain the complete molecular fragmentation mode, the data-related collection was also carried out. And sample information was collected when the mass/charge ratio of correction solution leucine enkephalin (LE) was 556.2771 (positive ion mode) and 554.2615 (negative ion mode) to ensure the accuracy of the collected results.

### 2.4 Establishment of a Chemical Compound Library for HJP

The chemical composition system information of the eight Chinese medicines in HJP was collected and sorted through online database platforms such as China National Knowledge Infrastructure (CNKI^1^), Traditional Chinese Medicine Systems Pharmacology Database and Analysis Platform (TCMSP^2^), the Encyclopedia of Traditional Chinese Medicine (ETCM^3^), Chem Spider^4^, and literature searches, etc. A self-built compound library containing compound name, molecular formula, chemical structure, and exact molecular weight was established by UNIFI software (Supplementary Table S1).

### 2.5 Data Analysis by UNIFI Platform

All MS data analysis was performed on the UNIFI software platform (Waters Corporation, Milford, United States). The acquisition time ranged from 1.5 min to 28.0 min. Quality inspection range: 50–1200 Da. The allowable range of quality error was within 5 ppm. The 2D peak intensity was set to a peak area greater than 100. The 3D peak intensity was greater than 200 for high-energy channels and greater than 300 for low-energy channels. The addition ions were +H, +Na (positive ion mode), and −H, +HCOO (negative ion mode). After all the data was analyzed and initially processed by the software, manual inspection and rechecking were still needed to investigate the chemical composition cracking pattern and enhance the accuracy of the characterization data.

### 2.6 Target Network Analysis

The targets were retrieved from the prediction of the online target platform Swiss Target Prediction^5^. “*Homo sapiens*” was the restrictor for target prediction. And the targets with a probability of more than 0.1 were collected. Meanwhile, the CVDs-related targets were obtained from the Gene cards database^6^. The Online Venn figure platform^7^ was used to get the Venn figure and HJP with CVDs intersection of targets. Cytoscape software (version 3.2.1) was applied to construct the chemical-target and protein-protein interactions (PPIs) networks. All proteins/genes were subjected to pathway enrichment analysis (GO and KEGG analysis) using the Metascape databases^8^. Those pathway terms with a *p*-value less than 0.05 were regarded as significant.

### 2.7 Molecular Docking

Download the protein PDB file of the core target from the RSCB PDB online platform^9^ and the MOL2 file of the core active ingredient from PubChem database^10^. Auto Dock Tools (1.5.6, The Scripps Research Institute) software was used for molecular docking, and the docking results were imported into Pymol (2.5.0, Schrodinger, Inc.) software for visualization. Finally, the binding strength and activity of the active ingredients and the target were evaluated according to the binding energy.

## 3 Results and Discussion

### 3.1 Identification and Characterization of Chemical Compounds

**FIGURE 1:**
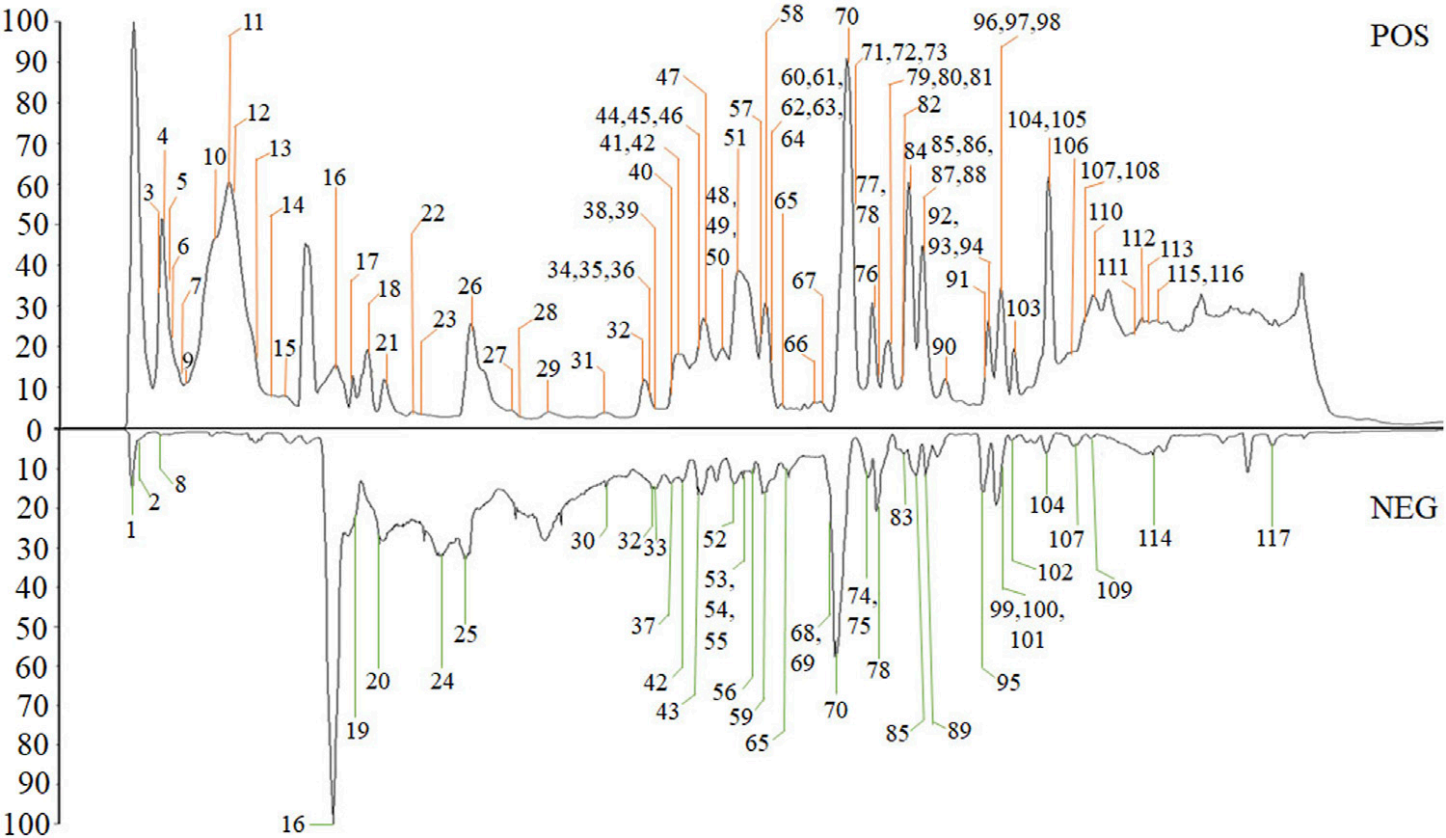
The TIC chromatograms of HJP from UPLC/Q-TOF-MS^E^ analysis.

**FIGURE 2:**
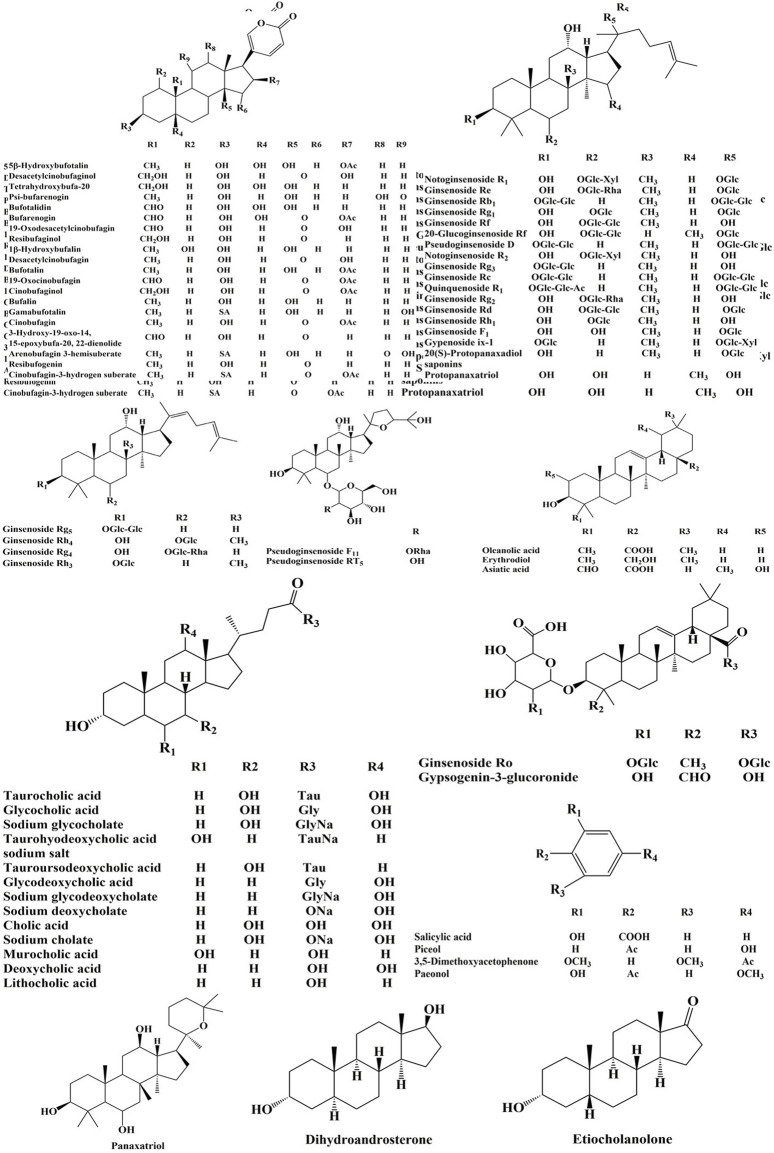
Chemical structures of compounds identified in HJP.

The high-resolution MS data of HJP were obtained by UPLC-Q-TOF/MS^E^. The total ion chromatography (TIC) of HJP in positive and negative ion modes is shown in Figure 1. The UNIFI screening platform was used to analyze the collected MS^E^ data, and the mass error and responsivity were investigated by automatically matching ion fragments. After standard comparison, literature review and further manual verification, a total of 117 compounds in HJP were identified or tentatively characterized. They were classified by ingredient sources, including 32 triterpenoid saponins, 13 bile acids, 22 bufadienolides, etc. Then, the detailed MS information of these components was summarized in Table 1. In addition, we tentative confirmed the structures of these compounds based on accurate mass, MS^E^ data, and related literature. The compounds with the same core structure were classified, such as dammarane type and oleanane type. Finally, the structure of the main chemical components in HJP is shown in Figure 2.

**TABLE 1:** Identification of chemical constituents of HJP by UPLC/Q−TOF−MS^E^.

No	Formular	t_R(min)_	Experimental mass (m/z)	Error (ppm)	MS and MS^E^ data (+ or −) (*m/z*)	Compound identification	Source
1	C_3_H_7_NO_2_	1.68	134.0456	−2.0	134.0456 [M + HCOO]^−^	Alanine	e, g, h
2	C_7_H_6_O_3_	1.86	137.024	−3.4	137.024 [M−H]^−^	Salicylic acid	a
					93.0343 [M−H−COO]^−^		
3	C_12_H_22_O_11_	2.02	365.1047	−1.9	365.1047 [M + H]^+^	Sucrose	a, b
					165.0777 [M + H−Glc−O]^+^		
4	C_6_H_14_N_4_O_2_	2.09	175.1193	2.3	175.1193 [M + H]^+^	Arginine	a, b, e, g, h
5	C_17_H_26_O_2_	2.37	263.1996	−3.5	263.1996 [M + H]^+^	Ginsenoyne I	a
					156.1021 [M + H−2H_2_O−C_5_H_11_]^+^		
6	C_8_H_8_O_2_	2.44	137.0603	4.2	137.0603 [M + H]^+^	Piceol	b
					119.051 [M + H−H_2_O]^+^		
					77.0391 [M + H−H_2_O−CH_3_CO]^+^		
7	C_9_H_11_NO_2_	2.51	180.1015	−2.1	180.1015 [M + H]^+^	Phenylalanine	e, g, h
					103.0548 [M−Ben]^+^		
					91.0549 [M−C_3_H_6_NO_2_]^+^		
					77.0391 [Ben]^+^		
8	C_10_H_12_O_3_	2.85	225.0775	3.0	225.0775 [M + HCOO]^−^	3,5-Dimethoxyacetophenone	b
9	C_19_H_26_O_3_	2.91	325.1763	−3.4	325.1763 [M + Na]^+^	Acetylpanaxydol	a
					286.1576 [M + H−H_2_O]^+^		
					195.1141 [M + H−H_2_O−CH_2_CO−CH_2_CH_3_−O]^+^		
10	C_12_H_16_N_2_O	3.3	205.1332	−1.7	205.1332 [M + H]^+^	Bufotenine	c
					146.0607 [M + H−C_3_H_9_N]^+^		
11	C_17_H_23_ClO_2_	3.84	317.1271	−2.6	317.1271 [M + Na]^+^	Ginsenoyne B	a
					89.0394 [M + H−2H_2_O−C_10_H_15_Cl]^+^		
12	C_13_H_18_N_2_O	3.9	219.1491	−0.2	219.1491 [M + H]^+^	Bufotenidine	c
					160.0758 [M + H−C_3_H_9_N]^+^		
13	C_27_H_46_O_2_	4.26	425.3391	0.3	425.3391 [M + Na]^+^	7β-Hydroxycholesterol	c
					407.3308 [M + H−H_2_O]^+^		
14	C_36_H_60_O_9_	4.36	637.4295	−2.4	637.4295 [M + H]^+^	Ginsenoside Rh_7_	a
					619.4187 [M + H−H_2_O]^+^		
					421.3459 [M + H−3H_2_O−Glc]^+^		
15	C_12_H_14_N_2_O	4.65	203.118	0.4	203.1181 [M + H]^+^	Dehydrobufotenine	c
					87.0866 [M−CH_3_]^+^		
16	C_47_H_80_O_18_	6.35	977.5309	−1.8	977.5309 [M + HCOO]^−^	*Notoginsenoside R_1_	b
					799.4813 [M−H−Xyl]^−^		
					637.4291 [M−H−Xyl−Glc]^−^		
					475.3743 [M−H−Xyl−2Glc]^−^		
					161.0429 [Glc−H]^−^		
17	C_26_H_36_O_7_	6.58	461.2534	0	461.2534 [M + H]^+^	5β-Hydroxybufotalin	c
					275.1335 [M + H−2H_2_O−α-pyr−CH_2_COO]^+^		
18	C_24_H_32_O_6_	6.74	417.2281	2.3	417.2281 [M + H]^+^	Desacetylcinobufaginol	c
					399.2173 [M + H−H_2_O]^+^		
19	C_48_H_82_O_18_	6.75	991.5471	−1.2	991.5471 [M + HCOO]^−^	*Ginsenoside Re	a
					783.486 [M−H−Rha−O]^−^		
					619.4148 [M−H−Rha−Glc−O]^−^		
20	C_42_H_72_O_14_	6.88	845.4891	−1.5	799.4821 [M−H]^−^	Pseudoginsenoside F_11_	a
					637.4300 [M−H−Rha−O]^−^		
					475.3755 [M−H−Rha−Glc−O]^−^		
21	C_15_H_22_	6.92	203.1797	1.4	203.1797 [M + H]^+^	Cuparene	b
					187.1485 [M−CH_3_]^+^		
					95.0865 [M−CH_3_−C_7_H_8_]^+^		
22	C_54_H_92_O_23_	7.96	1,125.6079	2.5	1,125.6079 [M + H]^+^	Ginsenoside Rb_1_	a
					801.4995 [M + H−2Glc]^+^		
23	C_24_H_34_O_6_	8.3	419.2421	−1.7	419.2421 [M + H]^+^	Tetrahydroxybufa-20	c
					347.2019 [M + H−4H_2_O]^+^		
					353.2111 [M + H−2H_2_O−CH_2_OH]^+^		
24	C_41_H_68_O_14_	8.98	829.456	−3.7	829.456 [M + HCOO]^−^	Ginsenoside Rg_8_	a
					499.2927 [M−H−2H_2_O−Rha−C_5_H_10_O_2_]^−^		
25	C_24_H_32_O_6_	9.13	461.2176	−1.1	461.2176 [M + HCOO]^−^	Psi-bufarenogin	c
					415.2116 [M−H]^−^		
					397.1994 [M−H−H_2_O]^−^		
					379.1873 [M−H−2H_2_O]^−^		
					287.1641 [M−H−H_2_O−α-pyr−CH_3_]^−^		
26	C_24_H_32_O_6_	9.23	417.2275	0.9	417.2275 [M + H]^+^	Bufotalidin	c
					439.2096 [M + Na]^+^		
					399.2173 [M + H−H_2_O]^+^		
					371.2211 [M + H−H_2_O−CO]^+^		
27	C_41_H_68_O_13_	10.13	783.4872	−2.1	783.4872 [M + H]^+^	Ginsenoside La	a
					441.3720 [M + H−H_2_O−2Glc]^+^		
					423.3610 [M + H−2H_2_O−2Glc]^+^		
28	C_30_H_48_O_3_	10.69	457.3666	−2.3	457.3666 [M + H]^+^	16-Oxoseratenediol	a
					439.3586 [M + H−H_2_O]^+^		
					421.3483 [M + H−2H_2_O]^+^		
29	C_9_H_10_O_3_	11.03	167.0708	3.5	167.0708 [M + H]^+^	Paeonol	a
					93.0347 [M + H−CH_3_CO−CH_3_O]^+^		
30	C_6_H_12_O_6_	12.06	534.1936	−0.8	534.1936 [M + HCOO]^−^	Galactose	a, h
					397.1625 [M−H−C_2_H_5_NO_3_]^−^		
31	C_24_H_32_O_6_	12.21	473.2178	1.7	473.2178 [M + H]^+^	Bufarenogin	c
					377.1715 [M + H−α-pyr]^+^		
					359.1651 [M + H−H_2_O−α-pyr]^+^		
32	C_26_H_45_NO_7_S	12.88	516.2979	−2	516.2979 [M + H]^+^	Taurocholic acid	g
					480.2805 [M + H−2H_2_O]^+^		
					462.2668 [M + H−3H_2_O]^+^		
					337.2517 [M + H−3H_2_O−Tau]^+^		
					126.0220 [Tau]^+^		
33	C_42_H_72_O_14_	12.97	845.4927	2.7	845.4927 [M + HCOO]^−^	Ginsenoside Rg_1_	a
					799.4867 [M−H]^−^		
					783.4883 [M−H−H_2_O]^−^		
					637.4105 [M−H−Glc]^−^		
					161.0436 [Glc]^−^		
34	C_24_H_30_O_6_	13.11	415.212	1.2	457.3679 [M + H]^+^	19-Oxodesacetylcinobufagin	c
					397.1994 [M + H−H_2_O]^+^		
					301.2499 [M + H−H_2_O−α-pyr]^+^		
35	C_30_H_48_O_3_	13.15	457.3679	0.6	457.3679 [M + H]^+^	Oleanolic acid	a, b
					439.3573 [M + H−H_2_O]^+^		
					421.3467 [M + H−2H_2_O]^+^		
36	C_42_H_72_O_14_	13.19	801.4996	0.1	823.4800 [M + Na]^+^	Ginsenoside Rf	a, b
					801.4996 [M + H]^+^		
					603.42470 [M + H−2H_2_O−Glc]^+^		
					585.4120 [M + H−3H_2_O−Glc]^+^		
37	C_26_H_43_NO_6_	13.34	478.3153	−4.4	478.3153 [M + HCOO]^−^	Glycocholic acid	e, g
					327.2627 [M−H−gly−2CH_3_]^−^		
					311.2639 [M−H−gly−2CH_3_−O]^−^		
38	C_24_H_32_O_5_	13.41	401.2326	1	401.2326 [M + H]^+^	Resibufaginol	c
					383.2170 [M + H−H_2_O]^+^		
					365.2065 [M + H−2H_2_O]^+^		
					347.1996 [M + H−3H_2_O]^+^		
39	C_36_H_62_O_10_	13.50	655.4413	−0.4	655.4413 [M + H]^+^	Pseudoginsenoside RT_5_	a
					603.4237 [M + H−2H_2_O−O]^+^		
					585.4097 [M + H−3H_2_O−O]^+^		
40	C_26_H_42_NNaO_6_	13.83	488.298	−0.6	488.2980 [M + H]^+^	Sodium glycocholate	e, g
					337.253 [M + H−Na−3H_2_O−Gly]^+^		
41	C_48_H_76_O_19_	13.86	979.4856	−1.7	979.4856 [M + Na]^+^	Ginsenoside Ro	a, b
					603.4229 [M + H−Glc−Glc-acid−H_2_O]^+^		
42	C_48_H_82_O_19_	13.89	985.5356	1.4	985.5356 [M + Na]^+^	20-Glucoginsenoside Rf	a
					766.4859 [M + H−Glc−H_2_O−O]^+^		
43	C_54_H_92_O_23_	14.16	1,107.5976	1.8	1,107.5976 [M−H]^−^	*Pseudoginsenoside D	a
					1,153.6048 [M + HCOO]^−^		
					945.5370 [M−H−Glc]^−^		
					783.4867 [M−H−2Glc]^−^		
					621.4408 [M−H−3Glc]^−^		
44	C_41_H_70_O_13_	14.2	771.4877	−1.5	771.4877 [M + H]^+^	Notoginsenoside R_2_	a
					605.4386 [M + H−H_2_O−Xyl−O]^+^		
					441.3715 [M + H−2H_2_O−Xyl−Glc]^+^		
					425.3778 [M + H−2H_2_O−Xyl−Glc−O]^+^		
45	C_42_H_72_O_13_	14.23	785.5029	−2.1	785.5029 [M + H]^+^	Ginsenoside Rg_3_	a, b
					605.4386 [M + H−H_2_O−Glc]^+^		
					459.3815 [M + H−2Glc]^+^		
					325.1133 [2Glc]^+^		
46	C_24_H_34_O_5_	14.44	403.2428	0.6	403.2428 [M + H]^+^	1β-Hydroxybufalin	c
					349.2166 [M + H−3H_2_O]^+^		
47	C_41_H_68_O_12_	14.5	753.4775	−1.1	753.4775 [M + H]^+^	Ginsenoside Rg_5_	a
					573.4102 [M + H−H_2_O−Glc]^+^		
48	C_24_H_32_O_5_	14.83	401.2325	0.7	401.2325 [M + H]^+^	Desacetylcinobufagin	c
					365.2127 [M + H−2H_2_O]^+^		
					347.2006 [M + H−3H_2_O]^+^		
49	C_36_H_60_O_8_	14.94	621.4366	0.8	621.4366 [M + H]^+^	Ginsenoside Rh_4_	a
					603.4243 [M + H−H_2_O]^+^		
					441.372 [M + H−H_2_O−Glc]^+^		
50	C_53_H_90_O_22_	14.95	1,079.5986	−1.0	1,079.5986 [M + H]^+^	Ginsenoside Rc	a, b
					1,104.7550 [M + Na]^+^		
					929.54 [M + H−H_2_O−Fur] ^+^		
51	C_26_H_36_O_6_	15.17	445.2588	0.6	445.2588 [M + H] ^+^	*Bufotalin	c
					467.2393 [M + Na] ^+^		
					409.2319 [M + H−2H_2_O]^+^		
					385.2365 [M + H−H_2_O−CH_2_CO]^+^		
					367.2268 [M + H−2H_2_O−CH_2_CO]^+^		
					349.2162 [M + H−α-pyr]^+^		
					331.1999 [M + H−H_2_O−α-pyr]^+^		
					271.2065 [M + H−2H_2_O−CH_2_CO−α-pyr]^+^		
52	C_56_H_94_O_24_	15.21	1,195.6054	−4.4	1,195.6054 [M + HCOO]^−^	Quinquenoside R_1_	a
					1,149.6032 [M−H]^−^		
					943.5289 [M−H−CH_3_CO−Glc]^−^		
					781.4687 [M−H−CH_3_CO−2Glc]^−^		
53	C_42_H_72_O_13_	15.23	807.485	−1.9	807.4850 [M + Na]^+^	Ginsenoside Rg_2_	a
					621.4353 [M + H−H_2_O−Rha]^+^		
					423.36173 [M + H−3H_2_O−Rha−Glc]^+^		
54	C_36_H_54_O_10_	15.26	647.3804	2.1	647.3804 [M + H]^+^	Gypsogenin-3-glucoronide	a
					385.2364 [M + H−GlcUA−C_6_H_12_]^+^		
					367.2268 [M + H−H_2_O−GlcUA−C_6_H_12_]^+^		
55	C_26_H_32_O_7_	15.41	457.2223	0.5	457.2223 [M + H]^+^	19-Oxocinobufagin	c
					439.3511 [M + H−H_2_O]^+^		
					332.1877 [M + H−α-pyr−CHO]^+^		
56	C_48_H_82_O_19_	15.64	991.5519	3.6	991.5519 [M + HCOO]^−^	*Ginsenoside Rd	a, b
					783.4906 [M−H−Glc]^−^		
					621.4358 [M−H−2Glc]^−^		
57	C_42_H_70_O_12_	15.68	767.4924	−2.1	767.4924 [M + H]^+^	Ginsenoside Rg_4_	a
					605.4392 [M + H−Rha−O]^+^		
					443.3875 [M + H−Rha−Glc−O]^+^		
58	C_18_H_32_O_16_	15.71	505.1781	3.5	505.1781 [M + H]^+^	Panose	a
					487.1651 [M + H−H_2_O]^+^		
					325.1127 [M + H−H_2_O−Glc]^+^		
					163.0503 [M + H−H_2_O−2Glc]^+^		
59	C_36_H_62_O_9_	15.76	683.4398	3.2	683.4398 [M + HCOO]^−^	Ginsenoside Rh_1_	a
					637.4327 [M−H]^−^		
					475.3783 [M−H−Glc]^−^		
					391.2803 [M−H−Glc−C_6_H_12_]^−^		
					161.0439 [Glc]^−^		
60	C_26_H_34_O_7_	15.79	459.2373	−0.9	459.2373 [M + H]^+^	Cinobufaginol	c
					381.2055 [M + H−H_2_O−α-pyr]^+^		
					363.1952 [M + H−α-pyr]^+^		
61	C_14_H_24_O	15.81	231.1718	−0.8	231.1718 [M + Na]^+^	1-Ethynylcyclododecanol	f
					213.1633 [M + H−H_2_O]^+^		
					147.1169 [M + H−H_2_O−C_3_H_7_]^+^		
62	C_36_H_62_O_9_	15.82	639.4436	−4.8	661.4256 [M + Na]^+^	Ginsenoside F_1_	a
					639.4436 [M + H]^+^		
					459.3836 [M + H−Glc−H_2_O]^+^		
					423.3620 [M + H−3H_2_O−Glc]^+^		
					405.3513 [M + H−4H_2_0−Glc]^+^		
63	C_15_H_24_O	15.85	221.1896	−1.6	221.1896 [M + H]^+^	Spathulenol	a, b
					203.1793 [M + H−H_2_O]^+^		
					187.1480 [M−H_2_O−CH_3_]^+^		
64	C_28_H_48_O	15.91	423.3617	4.6	423.3617 [M + Na]^+^	Campesterol	a
					383.2178 [M + H−H_2_O]^+^		
					311.2708 [M−H_2_O−C_5_H_11_]^+^		
65	C_26_H_44_NNaO_6_S	16.31	522.2867	1.5	522.2867 [M + H]^+^	Taurohyodeoxycholic acid sodium salt	e
					339.2702 [M + H−Na−2H_2_O−Tau]^+^		
66	C_47_H_80_O_17_	17.06	917.5485	1.8	939.5303 [M + Na]^+^	Gypenoside ix	b
					917.5485 [M + H]^+^		
					719.4701 [M + H−2H_2_O−Glc]^+^		
					587.4297 [M + H−2H_2_O−Xyl−Glc]^+^		
67	C_36_H_62_O_8_	17.11	645.4357	3.1	645.4357 [M + Na]^+^	20(S)-Protopanaxadiol saponins	a
					587.4297 [M + H−2H_2_O]^+^		
					425.3789 [M + H−2H_2_O−Glc]^+^		
					407.3684 [M + H−3H_2_O−Glc]^+^		
68	C_23_H_38_O_2_	17.39	345.2795	−1.1	345.2795 [M−H]^−^	5-Resorcinol	a
					205.1576 [M−H−C_10_H_20_]^−^		
69	C_24_H_40_O_5_	17.4	407.2798	−1.3	453.2850 [M + HCOO]^−^	*Cholic acid	e, g
					407.2798 [M−H]^−^		
					389.2656 [M−H−H_2_O]^−^		
					363.2880 [M−H−COO]^−^		
70	C_24_H_34_O_4_	17.49	387.2544	3.7	409.2367 [M + Na]^+^	*Bufalin	c
					387.2544 [M + H]^+^		
					369.2441 [M + H−H_2_O]^+^		
					351.2336 [M + H−2H_2_O]^+^		
					291.2113 [M + H−α-pyr]^+^		
					255.2109 [M + H−2H_2_O−α-pyr]^+^		
71	C_17_H_22_O_2_	17.66	259.169	−1.0	259.1690 [M + H]^+^	Ginsenoyne A	a
					227.1803 [M + H−2O]^+^		
					199.1488 [M + H−2O−C_2_H_4_]^+^		
72	C_15_H_18_	17.68	199.1488	3.2	199.1488 [M + H]^+^	Cadalene	b
					184.1220 [M + H-CH_3_]^+^		
					169.1018 [M + H-2CH_3_]^+^		
73	C_24_H_39_NaO_5_	17.71	431.2765	−0.6	431.2765 [M + H]^+^	Sodium cholate	g
					373.2751 [M + H−Na−2H_2_O]^+^		
					355.2647 [M + H−Na−3H_2_O]^+^		
					337.2539 [M + H−Na−4H_2_O]^+^		
					254.2035 [M + H−Na−3H_2_O−C_5_H_9_O_2_]^+^		
74	C_32_H_46_O_8_	17.91	557.3112	−1.4	557.3112 [M−H]^−^	Gamabufotalin-3-hydrogen suberate	c
					461.283 [M−H−α-pyr]^−^		
					383.2164 [M−H−SA]^−^		
					173.0804 [SA−H]^−^		
75	C_26_H_45_NO_6_S	17.96	500.3028	−2.5	500.3028 [M + H]^+^	Tauroursodeoxycholic acid	e, g
					339.268 [M + H−2H_2_O−Tau]^+^		
76	C_26_H_45_NO_6_	18	450.3213	−0.1	472.3027 [M + Na]^+^	Glycodeoxycholic acid	e, g
					450.3213 [M + H]^+^		
					339.2680 [M + H−2H_2_O−Gly]^+^		
					297.2643 [M + H−2H_2_O−Gly−CH_2_CO]^+^		
77	C2_6_H_42_NNaO_5_	18.21	472.3027	−1.3	472.3027 [M + H]^+^	Sodium glycodeoxycholate	e
					339.2683 [M + H−Na−2H_2_O−Gly]^+^		
					297.2643 [M + H−Na−2H_2_O−Gly−CH_2_CO]^+^		
78	C_36_H_60_O_7_	18.48	605.4411	−0.2	621.4298 [M + Na]^+^	Ginsenoside Rh_3_	a
					605.4411 [M + H]^+^		
					425.3780 [M + H−H_2_O−Glc]^+^		
					407.3673 [M + H−2H_2_O−Glc]^+^		
79	C_10_H_16_O	18.51	153.1277	2.3	153.1277 [M + H]^+^	Isopulegone	b
					123.1168 [M + H−2CH_3_]^+^		
80	C_19_H_32_O_2_	18.58	315.2297	0.8	315.2297 [M + Na]^+^	Dihydroandrosterone	f
					257.2265 [M + H−2H_2_O]^+^		
81	C_30_H_48_O_5_	18.61	489.3577	0.4	489.3577 [M + H]^+^	Asiatic acid	d
					471.3469 [M + H−H_2_O]^+^		
					453.3401 [M + H−2H_2_O]^+^		
					435.3232 [M + H−3H_2_O]^+^		
82	C_38_H_58_N_4_O_8_	18.82	699.4322	−0.7	699.4322 [M + H]^+^	Bufalitoxin	c
					681.4213 [M + H−H_2_O]^+^		
					351.2333 [M + H−H_2_O−Sub]^+^		
83	C_24_H_38_O_4_	18.94	389.2688	−2.3	435.2749 [M + HCOO]^−^	Diisooctyl phthalate	a
					389.2688 [M−H]^−^		
					374.2522 [M−H−CH_3_]^−^		
					332.2039 [M−H−C_4_H_9_]^−^		
84	C_26_H_34_O_6_	19.02	443.2428	0	465.2243 [M + Na]^+^	*Cinobufagin	c
					443.2428 [M + H]^+^		
					347.2005 [M + H−α−pyr]^+^		
					425.2307 [M + H−H_2_O]^+^		
					383.2217 [M + H−H_2_O−CH_2_CO]^+^		
					365.2111 [M + H−2H_2_O−CH_2_CO]^+^		
					337.2166 [M + H−2H_2_O−CH_2_CO−CO]^+^		
					319.2060 [M + H−3H_2_O−CH_2_CO−CO]^+^		
85	C_24_H_30_O_5_	19.26	399.2164	−0.5	399.2164 [M + H]^+^	3-Hydroxy-19-oxo-14, 15-epoxybufa-20,22-dienolide	c
					267.2266 [M + H−2O]^+^		
					271.2057 [M + H−α-pyr−2O]^+^		
86	C_32_H_44_O_9_	19.28	595.2865	−2.0	595.2865 [M + Na]^+^	Arenobufagin 3-hemisuberate	c
					399.2169 [M + H−SA]^+^		
					271.2057 [M + H−SA−α-pyr−2O]^+^		
87	C_24_H_32_O_4_	19.32	385.2372	−0.3	407.2186 [M + Na]^+^	*Resibufogenin	c
					385.2372 [M + H]^+^		
					253.1949 [M + H−2H_2_O−α-pyr]^+^		
88	C_10_H_16_	19.34	137.1327	1.8	137.1327 [M + H]^+^	Beta-pinene	b
					121.1014 [M−CH_3_]^+^		
					107.0861 [M + H−2CH_3_]^+^		
89	C_30_H_50_O_2_	19.55	443.3869	3.2	443.3869 [M + H]^+^	Erythrodiol	d
					425.3776 [M + H−H_2_O]^+^		
					407.3664 [M + H−2H_2_O]^+^		
90	C_19_H_34_O_2_	19.62	317.2438	−4	317.2438 [M + Na]^+^	Methyl linoleate	a
					137.1325 [M + H−C_9_H_18_O_2_]^+^		
91	C_24_H_39_NaO_4_	20.67	415.2822	0.8	415.2822 [M + H]^+^	Sodium deoxycholate	g
					339.2690 [M + H−2H_2_O−Na−OH]^+^		
92	C_19_H_30_O_2_	20.74	313.2139	0.2	313.2139 [M + Na]^+^	Etiocholanolone	f
					275.2003 [M−CH_3_]^+^		
					257.1884 [M−H_2_O−CH_3_]^+^		
93	C_38_H_56_O_4_	20.79	599.4081	1.7	599.40813 [M + Na]^+^	Campesteryl ferulate	a
					81.2788 [M + H−Ferulic-acid]^+^		
94	C_24_H_40_O_4_	20.85	393.3006	1.7	393.3006 [M + H]^+^	Murocholic acid	e
					375.2897 [M + H−H_2_O]^+^		
					357.2793 [M + H−2H_2_O]^+^		
					275.2003 [M + H−H_2_O−C_5_H_9_O−CH_3_]^+^		
95	C_24_H_40_O_4_	20.98	391.2849	−1.2	391.2849 [M−H]^−^	Deoxycholic acid	e, g
					373.2694 [M−H−H_2_O]^−^		
					355.2620 [M−H−2H_2_O]^−^		
96	C_16_H_28_O	21.02	237.2217	1.6	237.2217 [M + H]^+^	(1S,15S)-bicyclohexadecan	f
					81.0707 [M + H−C_10_H_19_O]^+^		
97	C_15_H_24_	21.05	227.1776	2.4	227.1776 [M + Na]^+^	α-Muurolene	a, b
					161.1329 [M + H−C_3_H_8_]^+^		
98	C_30_H_50_O_3_	21.06	459.3841	1.7	459.3841 [M + H]^+^	Dryobalanone	d
					441.3733 [M + H−H_2_O]^+^		
					423.3628 [M + H−2H_2_O]^+^		
					339.2693 [M + H−H_2_O−C_6_H_12_]^+^		
99	C_15_H_22_	21.08	203.1794	−0.2	203.1794 [M + H]^+^	α-Curcumene	a
					161.1329 [M + H−C_3_H_6_]^+^		
					147.1174 [M + H−C_4_H_8_]^+^		
100	C_30_H_52_O_4_	21.11	477.3951	2.7	477.3951 [M + H]^+^	Protopanaxatriol	a, b
					459.3841 [M + H−H_2_O]^+^		
					441.3733 [M + H−2H_2_O]^+^		
					423.3628 [M + H−3H_2_O]^+^		
101	C_20_H_40_N_2_O_8_	21.15	435.2772	−2.7	435.2772 [M−H]^−^	6-O-Hexonic acid	a
					389.2665 [M−H−H_2_O−CO]^−^		
102	C_53_H_92_O_7_	21.36	841.6895	−2.4	841.6895 [M + H]^+^	Sitoindoside Ⅱ	a
					703.5716 [M + H−3H_2_O−C_6_H_12_]^+^		
103	C_30_H_50_O_2_	22.20	443.3904	4.7	443.3904 [M + H]^+^	Dipterocarpol	d
					425.3778 [M + H−H_2_O]^+^		
104	C_12_H_25_NO	22.38	200.2004	−2.5	200.2004 [M + H]^+^	Dimethyldecanamide	a
					184.1692 [M−CH_3_]^+^		
105	C_15_H_26_O_2_	22.42	239.2	−2.2	239.2002 [M + H]^+^	4,10-Aromadendranediol	a
					221.2264 [M + H−H_2_O]^+^		
106	C_18_H_30_O_2_	22.78	279.2325	2.3	279.2325 [M + H] ^+^	Linolenic acid	a
					261.2206 [M + H−H_2_O]^+^		
					95.0863 [M + H−H_2_O−CH_2_CO−C_9_H_16_]^+^		
107	C_20_H_38_O_2_	22.86	311.2948	1.1	311.2948 [M + H]^+^	Eicosenoic acid	a
					250.1584 [M + H−H_2_O−CH2CO]^+^		
108	C_34_H_46_O_9_	22.90	599.3223	1.5	621.3052 [M + Na]^+^	Cinobufagin-3-hydrogen suberate	c
					599.3223 [M + H]^+^		
					425.2326 [M + H−SA]^+^		
109	C_42_H_82_NO_10_P	23.01	790.5596	−1.0	790.5596 [M−H]^−^	Phosphatidylserine	a
					480.3076 [M−H−CO_2_−C_18_H_34_O]^−^		
110	C_24_H_50_NO_7_P	23.2	496.34	0.5	496.3400 [M + H]^+^	Iysolecithin	g
					478.3293 [M + H−H_2_O]^+^		
					313.2740 [M + H−C_5_H_15_NO_4_P]^+^		
					184.0736 [M + H−C_19_H_37_O_3_]^+^		
111	C_16_H_25_N	24.08	232.2064	1.7	232.2064 [M + H]^+^	Muscopyridine	f
					215.1766 [M−CH_3_]^+^		
112	C_41_H_84_N_2_O_6_P	24.17	731.6041	−2.8	731.6041 [M + H]^+^	Sphingomyelin	g
					184.0732 [M + H−C_36_H_69_NO_2_]^+^		
113	C_30_H_52_O_4_	24.34	499.3753	−1.0	499.3753 [M + Na]^+^	Panaxatriol	b
					399.2156 [M + H−H_2_O−2CH_3_]^+^		
114	C_24_H_40_O_3_	24.37	375.2894	−3.0	375.2894 [M−H]^−^	Lithocholic acid	e, g
115	C_16_H_30_O_2_	25.00	277.2143	1.7	277.2143 [M + Na]^+^	13-Tetradecen acetate	a
116	C_20_H_32_O_2_	25.54	305.2467	−2.7	305.2467 [M + H]^+^	Arachidonate	a
					289.2157 [M−CH_3_]^+^		
					243.2094 [M + H−CH_3_−HCOO]^+^		
117	C_18_H_32_O_2_	27.13	279.2318	−4.0	279.2318 [M−H]^−^	Methyl heptadecadienoate	b
					179.1058 [M−H−C_7_H_16_]^−^		
					163.1109 [M−H−C_6_H_13_−OCH_3_]^−^		

Glc: glucose; Rha: rhamnose; Xyl: xylose; Gly: glycine; GlcUA: glucuronic acid; *α*-pyr: *α*-pyrone; Sub: suberylarginine; SA: suberic acid; Tau: taurine.

a Panax ginseng C. A. Meyer.

b Panax notoginseng (Burk.) F. H. Chen.

c Venenum Bufonis.

d Borneolum.

e Artificial Calculus Bovis.

f Artificial Moschus.

g Ox Bile Powder.

h Pearl.

*Identified by comparison with reference standards (Supplementary Figure S1).

### 3.2 Analysis of Chemical Component in HJP by UPLC/Q-TOF-MS^E^

#### 3.2.1 Triterpenoid Saponins

32 triterpenoid saponins were detected from HJP and were also the major bioactive constituents of *Panax ginseng C. A. Meyer* and *Panax notoginseng (Burk.) F. H. Chen*. These components have pharmacological effects such as cardiotonic, anti-oxidative stress, anti-myocardial ischemia, and immuno-enhancing function (Hu et al., 2018; Chen et al., 2020). The structure can also be divided into panaxadiol, panaxatriol, and oleanolic acid types. The primary fragmentation pattern of triterpenoid saponins was successive or simultaneous loss of the glycosidic unit at the site of C-20, C-3 or C-6 of ginsenosides until the formation of [Aglycon−H]^-^ ions. MS data analysis revealed the types and amount of glycosyl groups. For example, the mass differences of 162, 146 and 132Da suggested the presence of glucose (Glc), rhamnose (Rha), and xylose (Xyl), respectively (Fu et al., 2019).

**FIGURE 3:**
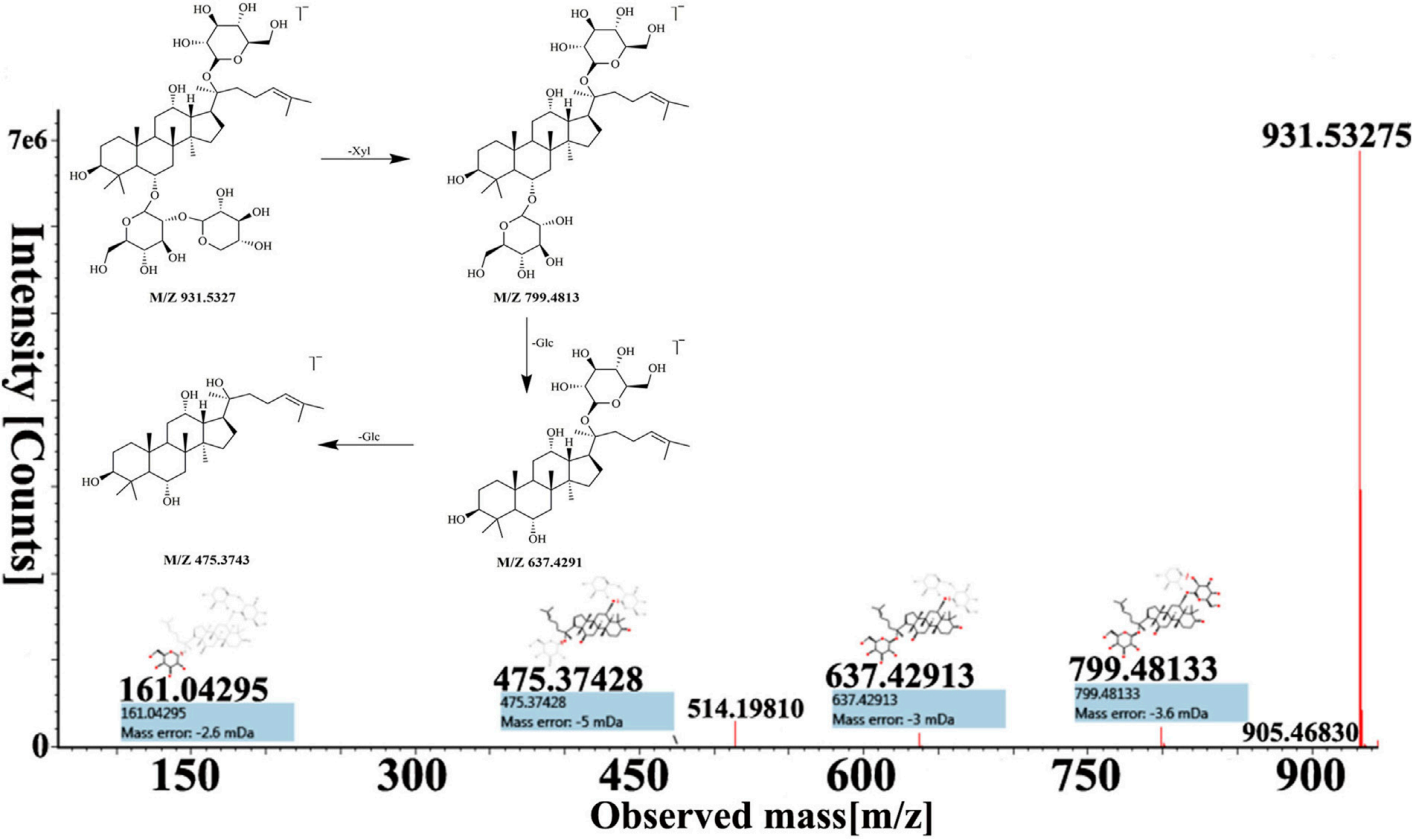
The MS spectra and fragmentation pattern of notoginsenoside R_1_ in negative ion mode.

Peak 16, peak 43, and peak 56 were identified as notoginsenoside R_1_, pseudoginsenoside D, and ginsenoside Rd by comparing retention time and fragmentation patterns with reference standards. We investigated the MS fragmentation pattern of notoginsenoside R_1_ in detail to facilitate the characterization of these ginsenosides (Figure 3). Notoginsenoside R_1_ revealed quasi-molecular ion [M−H]^-^ and [M + HCOO]^−^ at *m/z* 931.5327 and 977.5309 in negative ion mode. And then [M−H]^-^ ion lost a group of Xyl residue to form [M−H−Xyl]^−^ ion at *m/z* 799.4813. The following succession to lose Glc residue to form *m/z* 637.4291 [M−H−Xyl−Glc]^−^ and *m/z* 475.3743 [M−H−Xyl−2Glc]^−^ ions. Hence, it was tentatively characterized as notoginsenoside R_1_. In addition, the UNIFI software automatically matched the database with the acquisition mass spectrum data, and the compound was analyzed. Peak 36 had [M + H]^+^ and [M + Na]^+^ ions at *m/z* 801.4996 and 823.4800 in positive ion mode. The fragment ions of *m/z* 603.42470 and 585.4120 were formed under high collision energy. The mass error was within 0.10 ppm. The result of UNIFI platform matching for peak 36 was ginsenoside Rf. Through manual inspection and investigation of chemical structure, it is speculated that [M + H]^+^ ion loses two H_2_O and Glc residues, forming [M + H−2H_2_O−Glc]^+^ ion of *m/z* 603.4247. And then, it continued to lose the neutrality of H_2_O, which formed [M + H−3H_2_O−Glc]^+^ ion of *m/z* 585.4120. Therefore, the compound was identified as ginsenoside Rf, and its chemical structural formula was C_42_H_72_O_14_. Similarly, the matching result of compound 53 by UNIFI and manual verification shows ginsenoside Rg_2_.

#### 3.2.2 Cholic Acids

A total of 13 cholic acids were identified, which came from artificial *Calculus Bovis* and ox bile powder. These compounds have antipyretic, analgesic, cholagogic, anti-inflammatory, and detoxification effects (Xu et al., 2018; Floreani, 2020). They had a steroid parent nucleus in their structure and a pentanoic acid side chain at C-17. Due to the presence of polyhydroxy substituents and carboxyl groups in cholic acids, H_2_O was often lost during the cleavage process to produce [M + H−H_2_O]^+^ or [M + H−2H_2_O]^+^. In addition to the above rules, a neutral loss of 75 and 125Da may occur in the cleavage pathway for the conjugated bile acid formed by the amide bond of glycine or taurine to free bile acid, indicating the loss of glycine (Gly) and taurine (Tau), respectively.

**FIGURE 4:**
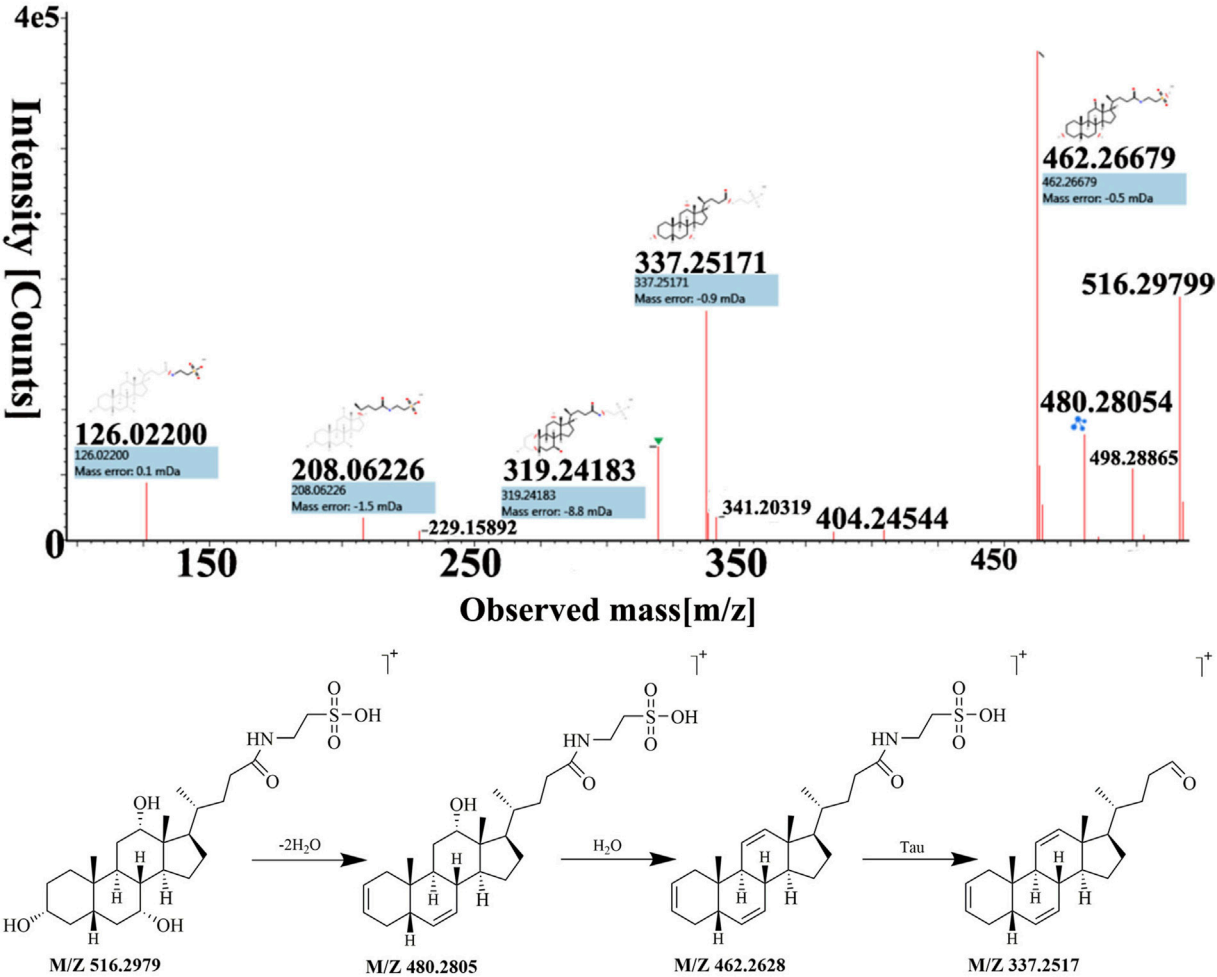
The MS spectra and fragmentation pattern of taurocholic acid in positive ion mode.

Peak 32 was unequivocally identified as taurocholic acid. The MS spectrum and possible fragmentation pattern of taurocholic acid were depicted in Figure 4. Taking taurocholic acid as an example, it produced fragment ions at *m/z* 516.2979 [M + H]^+^, 480.2805 [M + H−2H_2_O]^+^, 462.2628 [M + H−3H_2_O]^+^, and 337.2517 [M + H−3H_2_O−Tau]^+^. Moreover, peak 73 had a protonated [M + H]^+^ at *m/z* ion 431.2765 was identified as sodium cholate with a molecular formula of C_24_H_39_NaO_5_, and formed characteristic ions at *m/z* 373.2751 [M + H−Na−2H_2_O]^+^ and *m/z* 254.2035 [M + H−Na−3H_2_O−C_5_H_9_O_2_]^+^ through dehydration, sodium loss and C-17 valeric acid side chain dissociation. Their cleavage pathway conformed to the cleavage law of bile acids that were easy to lose H_2_O. We also used UNIFI software to analyze the unknown compounds and performed manual testing on the matching results. For instance, peak 40 exhibited [M + H]^+^ ion at *m/z* 488.2980, and UNIFI software speculated that its chemical formula was C_26_H_42_NNaO_6_. The manual analysis found it generated an [M + H−Na−3H_2_O−Gly]^+^ ion at *m/z* 337.2530 by the loss of Na^+^, 3H_2_O and Gly residue. Thus, peak 40 was assigned as sodium glycocholate. Similarly, through UNIFI platform matching, the preliminary results showed that the peak 95 is deoxycholic acid.

#### 3.2.3 Bufadienolides

22 bufadienolides were identified in HJP, which were derived from *Venenum Bufonis*. It has a strong cardiac excitatory effect like that of digitalis. Importantly, the drug possessed many advantages such as quick-acting, no accumulation and diuretic action (Wansapura et al., 2009; Xie et al., 2016; Zhang et al., 2019a). These components have a higher response in the positive ion mode, and there were [M + H]^+^ quasi-molecular ions. Due to the polyhydroxy and *α*-pyrone (α-pyr) in its structure, it was easy to lose H_2_O (18 Da) and *α*-pyr (96 Da) residue under high collision energy in mass spectrometry. In addition, deacetylation caused a neutral loss of 60 Da.

**FIGURE 5:**
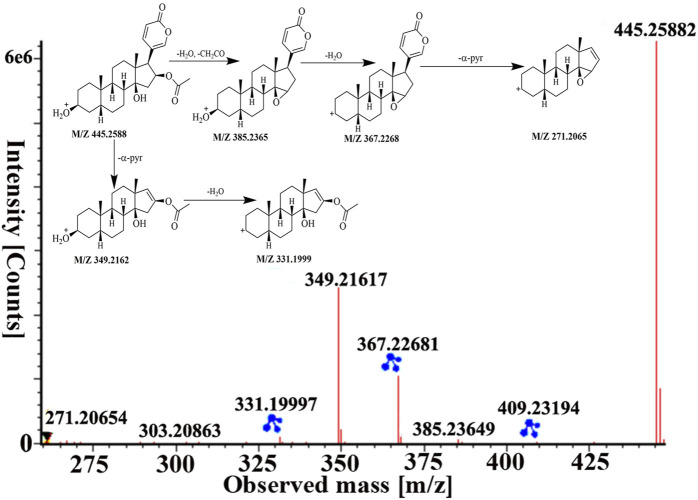
The MS spectra and fragmentation pathway of bufotalin in positive ion mode.

Peaks 51 was identified as bufotalin, which belongs to bufadienolides, based on retention time and fragment behavior of reference standard. Bufotalin showed [M + H]^+^ and [M + Na]^+^ ions at *m/z* 445.2588 and 467.2393. Next, it underwent deacetylations and dehydrations to form [M + H−H_2_O−CH_2_CO]^+^ and [M + H−2H_2_O−CH_2_CO]^+^ fragment ions at *m/z* 385.2365 and 367.2268. And then, dissociation of the *α*-pyran ring gave rise to [M + H−2H_2_O−CH_2_CO−α-pyr]^+^ ion at *m/z* 271.2065. Furthermore, it directly lost the *α*-pyr and then lost H_2_O to obtain fragment ions of *m/z* 349.2162 and 331.1999, respectively. The high-energy MS spectra and the proposed fragment pathway of bufotalin are depicted in Figure 5. Peaks 60, 70, 84, and 87 have similar cleavage rules of bufotalin, and all have the neutral loss of the characteristic fragment ions of the *α*-pyrone. By matching with UNIFI software and comparing with standard products, they were identified as cinobufaginol, bufalin, cinobufagin, and resibufogenin.

#### 3.2.4 Others

In addition to the three major components, we also identified indole alkaloids (3), amino acids (4), organic acids (6), polysaccharides (3), lecithins (2), and volatile oils (23). These compounds showed higher responses in the positive ion mode, in the form of [M + H]^+^ ions. And there were usually contains hydroxyl groups and other easy-to-lost groups in the structure of these components, which generated [M + H−H_2_O]^+^ or [M + H−2H_2_O]^+^ ions, etc. at high collision energies. Taking peaks 80 and 89 as examples, peak 80 showed [M + H]+and [M + Na]^+^ ions at *m/z* 293.2557 and 315.2297. The mass error range was within 0.8 ppm. It continuously lost two H_2_O under high collision energy and obtained [M + H−2H_2_O]^+^ ion with *m/z* 257.2265, identified as dihydroandrosterone. Similarly, peak 89 has an [M + H]^+^ ion of *m/z* 443.3869 in the positive ion mode. Then, by continuously losing H_2_O [M + H−H_2_O]^+^ and [M + H−2H_2_O]^+^ fragment examples were formed at *m/z* 425.3776 and 407.3664, and the compound was finally identified as erythrodiol.

### 3.3 Network Pharmacology Research

#### 3.3.1 Target Prediction and Screening

Generally speaking, molecules with oral bioavailability (OB) ≥ 30% and drug-like properties (DL) ≥ 0.18 are considered to have better pharmacological effects (Wei et al., 2020). We reference the OB and DL values combined with Swiss ADME (http://www.swissadme.ch/) to screen the ingredients with high gastrointestinal absorption, and a total of 26 active ingredients were obtained (Table 2). Then, the disease-related target proteins and gene targets were obtained through the Swiss Target Prediction, Gene Cards, Uniprot, and OMIM database. A total of 276 drug-related and 1,293 CVDs-related targets were obtained.

**TABLE 2:** Active ingredients list of HJP.

Name	Compound	Chemical formula	Source
RS1	Ginsenoside Rg_3_	C_42_H_72_O_13_	a
RS2	Ginsenoside Rc	C_53_H_90_O_22_	a
RS3	Ginsenoside Rh_1_	C_35_H_60_O_9_	a
RS4	Ginsenoside Rg_2_	C_42_H_72_O_13_	a
RS5	Ginsenoside Rb_1_	C_54_H_92_O_23_	a
RS6	Ginsenoside Rf	C_42_H_72_O_14_	a
RS7	Ginsenoside Rg_1_	C_42_H_72_O_14_	a
RS8	Ginsenoside Re	C_48_H_82_O_18_	a
CS1	5β-Hydroxybufotalin	C_26_H_36_O_7_	b
CS2	7β-Hydroxycholesterol	C_27_H_46_O_2_	b
CS3	19-Oxo-cinobufagin	C_26_H_32_O_7_	b
CS4	19-Oxo-desacetylcinobufagin	C_24_H_30_O_6_	b
CS5	Bufotenidine	C_13_H_18_N_2_O	b
CS6	Bufotenine	C_12_H_16_N_2_O	b
CS7	Psi-bufarenogin	C_24_H_32_O_6_	b
CS8	Bufarenogin	C_24_H_32_O_6_	b
CS9	Resibufogenin	C_24_H_32_O_4_	b
BP1	Asiatic acid	C_30_H_48_O_5_	c
RGNH1	Murocholic acid	C_24_H_40_O_4_	d
A1	Cholic acid	C_24_H_40_O_5_	d, e
A2	Deoxycholic acid	C_24_H_40_O_4_	d, e
A3	Lithocholic acid	C_24_H_40_O_3_	d, e
A4	Chenodeoxycholic acid	C_24_H_40_O_4_	d, e
A5	Sodium glycocholate	C_26_H_42_NNaO_6_	d, e
A6	Sodium deoxycholate	C_24_H_39_NaO_4_	d, e
A7	Glycocholic Acid	C_26_H_43_NO_6_	d, e

a Panax ginseng C. A. Meyer.

b Venenum Bufonis.

c Borneolum.

d Artificial Calculus Bovis.

e Ox Bile Powder.

#### 3.3.2 Construction of D-I-T and PPI Network

**FIGURE 6:**
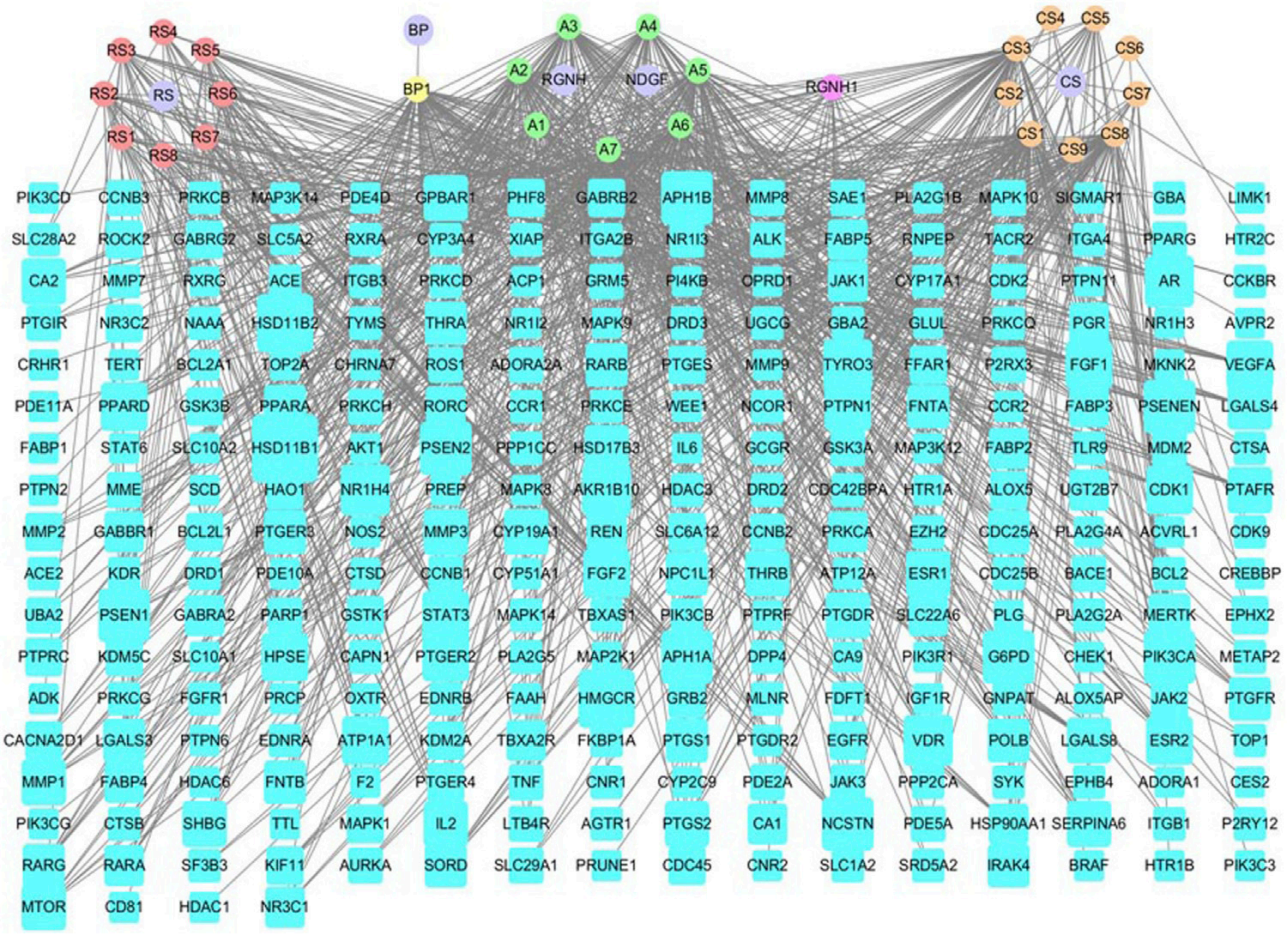
Drug-ingredient-target network of HJP (RS: *Panax ginseng C. A. Meyer.* CS: *Venenum Bufonis.* BP: *Borneolum.* RGNH: Artificial Calculus Bovis. NDGF: Ox Bile Powder. The purple round nodes were composed of drugs surrounded by their particular ingredients. The blue rectangular node represented the gene targets.).

**FIGURE 7:**
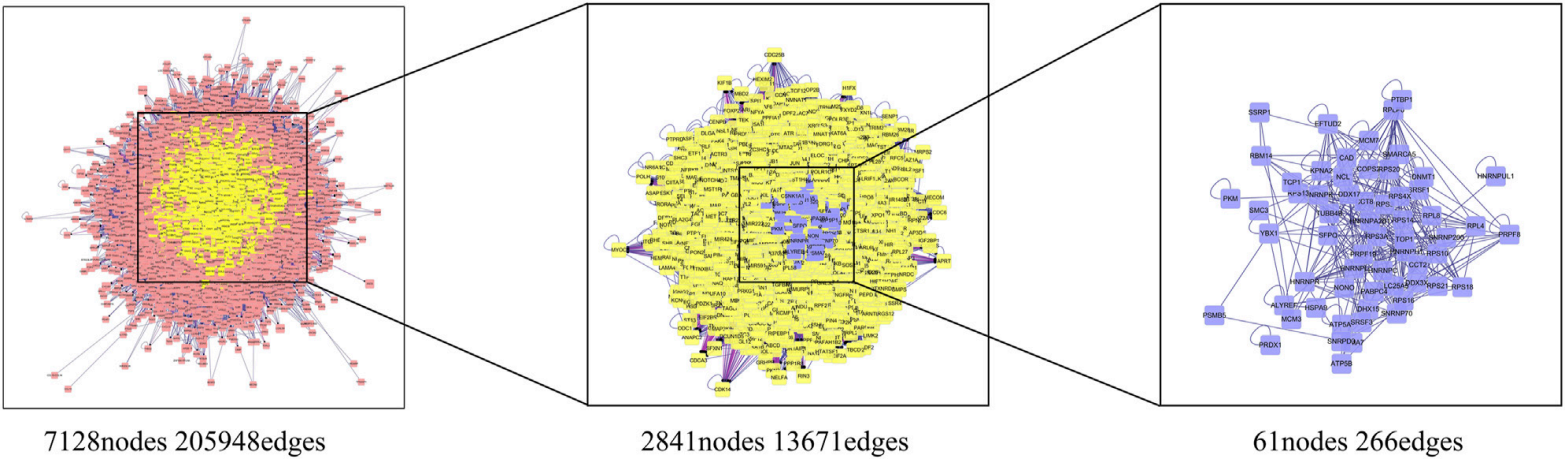
Protein-protein interaction (PPI) network results for CVDs treatment of HJP.

The drug-ingredient-target (D-I-T) network contains 308 nodes, including six drugs, 26 active compounds, and 276 targets (Figure 6). The ingredient that corresponded to more targets meant more importance. According to the degree analysis, the top three compounds were 5β-hydroxybufotalin (CS1), 19-oxo-cinobufagin (CS3), and bufarenogin (CS8). In addition, we imported drug-related targets and CVDs-related targets into Cytoscape 3.7.2 software to build the protein-protein interaction (PPI) Network (Figure 7). The PPI network has 61 nodes and 266 edges.

#### 3.3.3 GO and KEGG Pathway Enrichment Analysis

**FIGURE 8:**
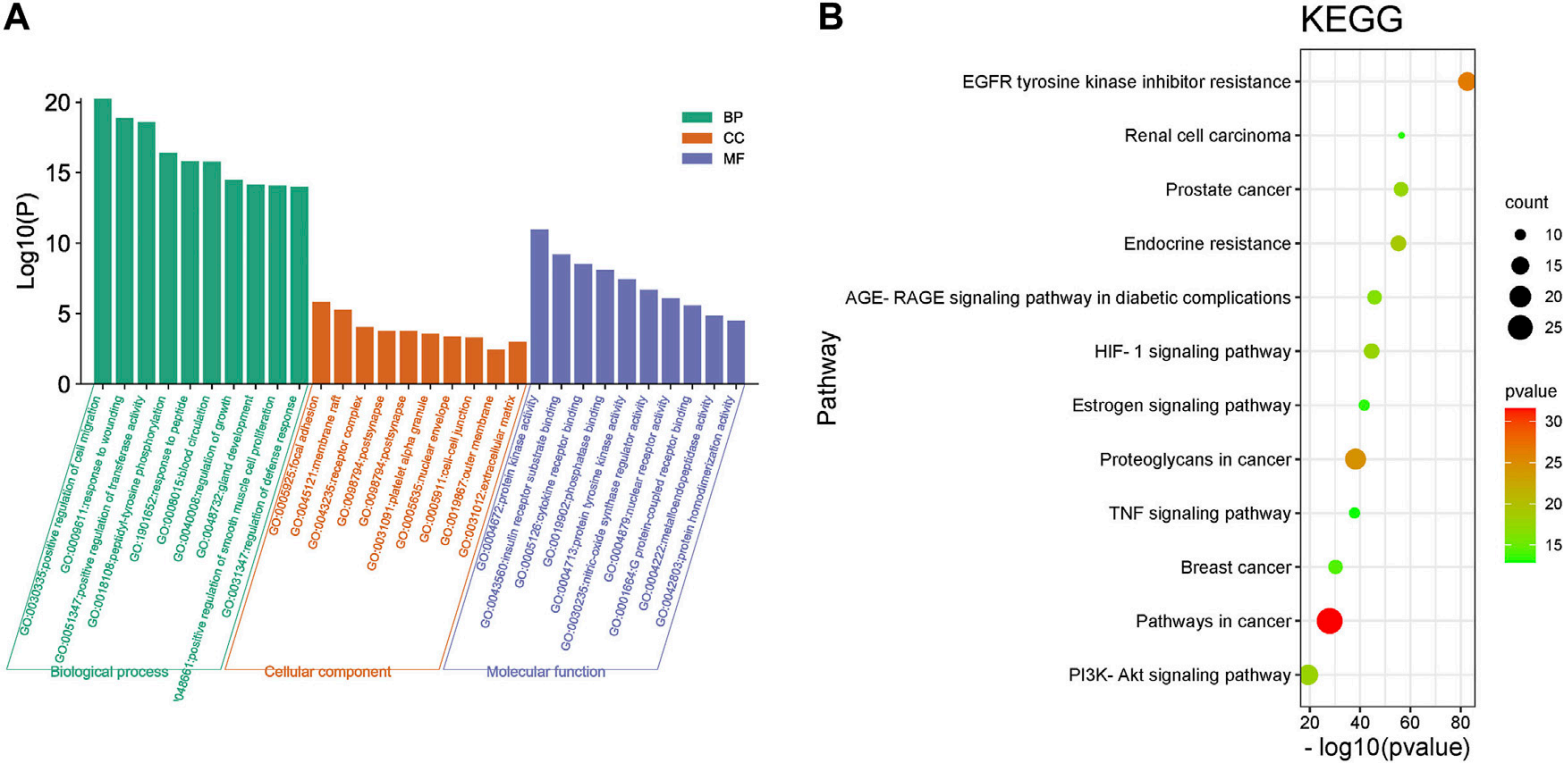
GO **(A)** and KEGG pathway **(B)** enrichment analysis of results for CVD treatment of HJP.

The Gene Ontology (GO) function enrichment analysis results of HJP for the treatment of CVDs core target genes are shown in Figure 8A. The biological processes mainly included positive regulation of cell migration, response to wounding, blood circulation, and positive regulation of cell migration. The main targets of cell component analysis were focal adhesion, membrane raft, receptor complex, nuclear envelope, etc. Simultaneously, molecular function terms mainly contained protein kinase activity, receptor substrate binding, cytokine receptor binding, phosphatase binding, and so on.

A total of 132 signal pathways were enriched, and 20 top-ranking pathways were screened out to analyze (*p* < 0.05). Kyoto Encyclopedia of Genes and Genomes (KEGG) pathway enrichment analysis results showed that the targets of HJP for the treatment of CVDs mainly focused on endocrine resistance, PI3K-Akt signaling pathway, and HIF-1 signaling pathway, etc. (Figure 8B).

#### 3.3.4 Component-Target-Pathway (C-T-P) Network Construction

**FIGURE 9:**
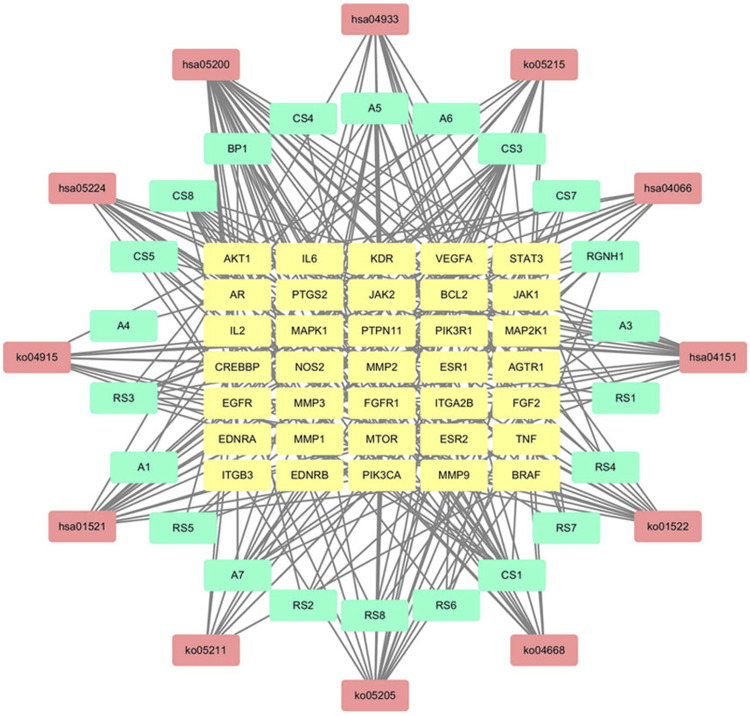
C-T-P network for CVDs treatment of HJP (The yellow, blue and red nodes represented the active components, gene targets, and pathways, respectively.).

The Merge function of Cytoscape 3.7.2 software was used to combine the effective compounds of HJP, the action targets of compounds, and the signal pathways. Afterward, visual analysis was performed to construct an overall network diagram of C-T-P (Figure 9). As we know, the more connections you have, the more critical it is. The results showed that 5β-hydroxybufotalin, 19-oxo-cinobufagin, and bufarenogin were the more essential ingredients. Besides, the active ingredients played a role in CVDs treatment mainly by acting on PIK3CA, MAPK1, MTOR, VEGFA, and other targets.

#### 3.3.5 Results of Molecular Docking

**FIGURE 10:**
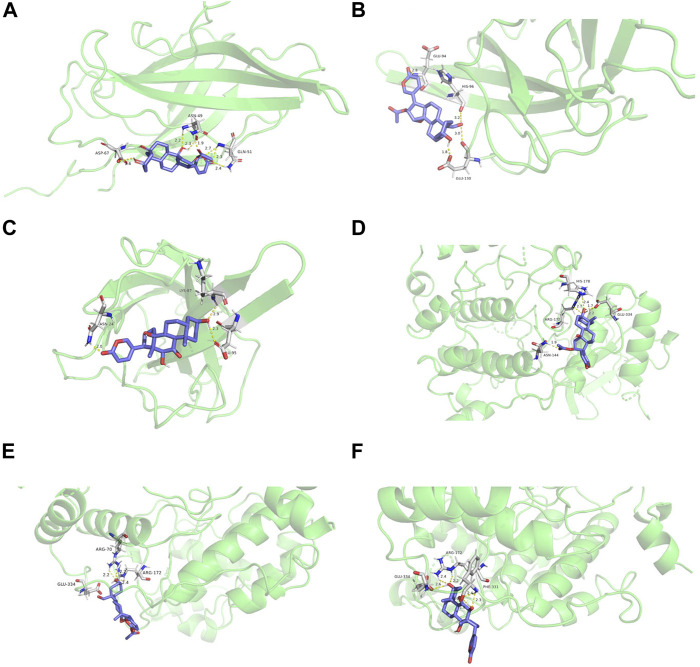
Molecular docking structure diagram **(A)**. PIK3CA and 5β-hydroxybufotalin **(B)**. PIK3CA and 19-oxo-cinobufagin **(C)**. PIK3CA and bufarenogin **(D)**. MAPK1 and 5β-hydroxybufotalin **(E)**. MAPK1 and 19-oxo-c.

We performed flexible molecular docking between the top three core active ingredients and the top two core targets. Docking binding energy below 0 indicates that two molecules can spontaneously bind, and smaller binding energy leads to a more stable conformation. Therefore, we can conclude that the core active ingredients have a good affinity for the target proteins. Furthermore, chemical components acting on multiple amino acid residues also suggest the multi-target characteristics of TCM preparation. The docking energy and its local structure are shown in Table 3 and Figure 10.

**TABLE 3:** Molecular docking parameter table.

Target protein	Docking parameters	5β-Hydroxybufotalin	19-Oxo-cinobufagin	Bufarenogin
PIK3CA	binding energy/kJ·mol^−1^	−6.01	−5.97	−5.83
	Participating amino acid residues	ASP-67, ASN-49, GLN-51	GLU-94, HIS-96, GLU-130	LYS-87, GLU-95, ASN-24
MAPK1	binding energy/kJ·mol^−1^	−6.01	−6.68	−6.13
	Participating amino acid residues	HIS-178, ARG-172, ASN-144, GLU-334	ARG-70, ARG-172, GLU-334	GLU-334, ARG-172, PHE-331

## 4 Discussion

HJP is a TCM preparation widely used to treat CVDs. However, only preliminary studies have been conducted on HJP. There were still gaps in the research on the material basis and mechanism of action of HJP in CVDs treatment. It restricted the further clinical research and market promotion of HJP. Therefore, this research has developed a comprehensive research strategy combining UPLC-Q-TOF/MS^E^ and network pharmacology to fill these existing gaps. Finally, this research identified and initially characterized 117 compounds, including triterpenoid saponins (32), cholic acids (13), bufadienolides 22) and others. It can be considered that the established UPLC-Q-TOF/MS^E^ method was more comprehensive for the detection and identification of the chemical components in HJP. The research results provided more basic information on chemical substances for the further research of HJP.

According to the constructed C-T-P network analysis, 5β-hydroxybufotalin, 19-oxo-cinobufagin, and bufarenogin were the active ingredients associated with the most targets. The results of molecular docking also verified that they have good binding properties with target genes. In the current study, the results of GO and pathway enrichment analysis showed that HJP treated CVDs, which may involve the following biological processes and pathways: receptor complex, receptor substrate binding, cytokine receptor binding, endocrine resistance, PI3K-Akt signaling pathway, HIF-1 signaling pathway, and so on. According to the matching results, the CVDs-related targets of HJP were PIK3CA, MAPK1, MTOR, VEGFA, and EGFR, etc. In the following research, we will select some key targets to explore the possible mechanism of HJP in CVDs treatment.

HJP may treat CVDs by modulating the mechanism of angiogenesis. Angiogenesis is responsible for a wide variety of physio/pathological processes, including CVDs. It is strictly controlled by the balance of pro-angiogenic and anti-angiogenic factors, such as vascular endothelial growth factor (VEGF) and epidermal growth factor (EGF), etc. (Ambasta et al., 2011). Studies have shown that bufalin can target the mTOR/VEGF signaling pathway, affecting the tumor vascular microenvironment and improving the anti-angiogenic effect (Wang et al., 2018a). Gamabufotalin, an important active compound of bufadienolides, was also proved as a potential anti-angiogenesis agent that targets VEGF/VEGFR-2 signaling pathways (Tang et al., 2016). Furthermore, the PIK3CA gene can increase the release of NO through the PI3K/Akt/e-NOS pathway and induce the regeneration of myocardial ischemic blood vessels (Zhang et al., 2019b). In HJP, 16 compounds, including 5β-hydroxybufotalin and bufarenogin, have been shown to have effects on PIK3CA and VEGFA by the C-T-P network.

On the other hand, HJP can regulate inflammation and oxidative stress targets to treat CVDs. The most promising intervention direction for atherosclerosis is lipid metabolism and inflammation. And inflammatory factors are involved in the occurrence and development of CVDs. For instance, MAPK1 is involved in various physiological and pathological processes, such as the body’s adaptive response to external environmental stress and inflammation. It is the common target of many anti-inflammatory drugs and can promote the production of IL-β and TNF-α, pro-inflammatory cytokines that can activate enzymes related to inflammation. In addition, studies have found that Ginsenoside Re is a powerful antioxidant protecting cardiomyocytes from oxidation by scavenging free radicals (Wang et al., 2018b). And Taurodeoxycholic acid can regulate the MAPK and NF-KB pathways and promote the anti-inflammatory cytokine IL-10 (Ko et al., 2017). Glycocholic acid and glycosyllithocholic acid can reduce the synthesis and release of nitric oxide, inhibit the production of leukotriene B_4_ and prostaglandin E_2_, and produce anti-inflammatory effects (Li, 2006). These selected active ingredients in HJP played an important role in treating CVDs and provide a reference for more in-depth material basic research.

## 5 Conclusion

This study used integrated research on chemical profiling to explore the chemical constituents and action mechanism of HJP by UPLC-Q-TOF/MS^E^ and network pharmacology. Finally, on the basis of the research on the chemical composition of HJP, we initially revealed that HJP might exert therapeutic effects on CVDs by regulating the mechanism of angiogenesis, inflammation, and oxidative stress targets.

On the one hand, this paper provides a certain basis and reference for the further study of the pharmacodynamic material basis and *in vivo* mechanism of HJP. On the other hand, the integrated methods and results it established also provide some reference for other basic research on TCM. However, we contrast the network pharmacology research content in this paper with the latest Network pharmacology evaluation method guidance (Li et al., 2021). It was found that the verification experiment used a combination of computer-aided and literature data. Although the overall experiment satisfies the standardization and rationality, the reliability has certain limitations. Therefore, in the future, we will use the Network pharmacology evaluation method guidance as a guide to experimentally verify the results to further increase their reliability.

## Data Availability

The datasets presented in this study can be found in online repositories. The names of the repository/repositories and accession number(s) can be found in the article/Supplementary Material.

## Footnotes

1
https://www.cnki.net/.

2
https://old.tcmsp-e.com/tcmsp.php.

3
http://www.tcmip.cn/ETCM/index.php/Home/Index/.

4
http://www.chemspider.com/.

5
http://old.swisstargetprediction.ch/.

6
https://www.genecards.org/.

7
http://bioinformatics.psb.ugent.be/webtools/Venn/.

8
https://metascape.org/gp/index.html#/main/step1.

9
https://www1.rcsb.org/.

10
https://pubchem.ncbi.nlm.nih.gov/.
