# Supplementary material for: The Integrated Study on the Chemical Profiling to Explore the Constituents and Mechanism of Traditional Chinese Medicine Preparation Huatuo Jiuxin Pills Based on UPLC-Q-TOF/MSE and Network Pharmacology
Source: Front Mol Biosci. 2022 Mar 31;9:818285. doi: 10.3389/fmolb.2022.818285 (PMC9008511; doi:10.3389/fmolb.2022.818285)
Supplement: Supplementary file 1 [file Table2.docx]

SupplementaryTable 2

Identification of chemical constituents of HJP by UPLC/Q−TOF−MS^E^

| No. | Formular | t_R(min)_ | Experimental mass (m/z) | Error  (ppm) | MS and MS^E^ data (+ or −) (*m/z*) | Compound identification | Source |
| --- | --- | --- | --- | --- | --- | --- | --- |
|  | C_3_H_7_NO_2_ | 1.68 | 134.0456 | −2.0 | 134.0456[M+HCOO]^−^ | Alanine | e, g, h |
|  | C_7_H_6_O_3_ | 1.86 | 137.024 | −3.4 | 137.024[M−H]^−^  93.0343[M−H−COO]^−^ | Salicylic acid | a |
|  | C_12_H_22_O_11_ | 2.02 | 365.1047 | −1.9 | 365.1047[M+H]^+^  165.0777[M+H−Glc−O]^+^ | Sucrose | a, b |
|  | C_6_H_14_N_4_O_2_ | 2.09 | 175.1193 | 2.3 | 175.1193[M+H]^+^ | Arginine | a, b, e, g, h |
|  | C_17_H_26_O_2_ | 2.37 | 263.1996 | −3.5 | 263.1996[M+H]^+^  156.1021[M+H−2H_2_O−C_5_H_11_]^+^ | Ginsenoyne I | a |
|  | C_8_H_8_O_2_ | 2.44 | 137.0603 | 4.2 | 137.0603[M+H]^+^  119.051[M+H−H_2_O]^+^  77.0391[M+H−H_2_O−CH_3_CO]^+^ | Piceol | b |
|  | C_9_H_11_NO_2_ | 2.51 | 180.1015 | −2.1 | 180.1015[M+H]^+^  103.0548[M−Ben]^+^  91.0549[M−C_3_H_6_NO_2_]^+^  77.0391[Ben]^+^ | Phenylalanine | e, g, h |
|  | C_10_H_12_O_3_ | 2.85 | 225.0775 | 3.0 | 225.0775[M+HCOO]^−^ | 3,5-Dimethoxyacetophenone | b |
|  | C_19_H_26_O_3_ | 2.91 | 325.1763 | −3.4 | 325.1763[M+Na]^+^  286.1576[M+H−H_2_O]^+^  195.1141[M+H−H_2_O−CH_2_CO−CH_2_CH_3_−O]^+^ | Acetylpanaxydol | a |
|  | C_12_H_16_N_2_O | 3.3 | 205.1332 | −1.7 | 205.1332[M+H]^+^  146.0607[M+H−C_3_H_9_N]^+^ | Bufotenine | c |
|  | C_17_H_23_ClO_2_ | 3.84 | 317.1271 | −2.6 | 317.1271[M+Na]^+^  89.0394[M+H−2H_2_O−C_10_H_15_Cl]^+^ | Ginsenoyne B | a |
|  | C_13_H_18_N_2_O | 3.9 | 219.1491 | −0.2 | 219.1491[M+H]^+^  160.0758[M+H−C_3_H_9_N]^+^ | Bufotenidine | c |
|  | C_27_H_46_O_2_ | 4.26 | 425.3391 | 0.3 | 425.3391[M+Na]^+^  407.3308[M+H−H_2_O]^+^ | 7β-Hydroxycholesterol | c |
|  | C_36_H_60_O_9_ | 4.36 | 637.4295 | −2.4 | 637.4295[M+H]^+^  619.4187[M+H−H_2_O]^+^  421.3459[M+H−3H_2_O−Glc]^+^ | Ginsenoside Rh_7_ | a |
|  | C_12_H_14_N_2_O | 4.65 | 203.118 | 0.4 | 203.1181[M+H]^+^  87.0866[M−CH_3_]^+^ | Dehydrobufotenine | c |
|  | C_47_H_80_O_18_ | 6.35 | 977.5309 | −1.8 | 977.5309[M+HCOO]^−^  799.4813[M−H−Xyl]^−^  637.4291[M−H−Xyl−Glc]^−^  475.3743[M−H−Xyl−2Glc]^−^  161.0429[Glc−H]^−^ | *Notoginsenoside R_1_ | b |
|  | C_26_H_36_O_7_ | 6.58 | 461.2534 | 0 | 461.2534[M+H]^+^  275.1335[M+H−2H_2_O−α-pyr−CH_2_COO]^+^ | 5β-Hydroxybufotalin | c |
|  | C_24_H_32_O_6_ | 6.74 | 417.2281 | 2.3 | 417.2281[M+H]^+^  399.2173[M+H−H_2_O]^+^ | Desacetylcinobufaginol | c |
|  | C_48_H_82_O_18_ | 6.75 | 991.5471 | −1.2 | 991.5471[M+HCOO]^−^  783.486[M−H−Rha−O]^−^  619.4148[M−H−Rha−Glc−O]^−^ | *Ginsenoside Re | a |
|  | C_42_H_72_O_14_ | 6.88 | 845.4891 | −1.5 | 799.4821[M−H]^−^  637.4300[M−H−Rha−O]^−^  475.3755[M−H−Rha−Glc−O]^−^ | Pseudoginsenoside F_11_ | a |
|  | C_15_H_22_ | 6.92 | 203.1797 | 1.4 | 203.1797[M+H]^+^  187.1485[M−CH_3_]^+^  95.0865[M−CH_3_−C_7_H_8_]^+^ | Cuparene | b |
|  | C_54_H_92_O_23_ | 7.96 | 1125.6079 | 2.5 | 1125.6079[M+H]^+^  801.4995[M+H−2Glc]^+^ | Ginsenoside Rb_1_ | a |
|  | C_24_H_34_O_6_ | 8.3 | 419.2421 | −1.7 | 419.2421[M+H]^+^  347.2019[M+H−4H_2_O]^+^  353.2111[M+H−2H_2_O−CH_2_OH]^+^ | Tetrahydroxybufa-20 | c |
|  | C_41_H_68_O_14_ | 8.98 | 829.456 | −3.7 | 829.456[M+HCOO]^−^  499.2927[M−H−2H_2_O−Rha−C_5_H_10_O_2_]^−^ | Ginsenoside Rg_8_ | a |
|  | C_24_H_32_O_6_ | 9.13 | 461.2176 | −1.1 | 461.2176[M+HCOO]^−^  415.2116[M−H]^−^  397.1994[M−H−H_2_O]^−^  379.1873[M−H−2H_2_O]^−^  287.1641[M−H−H_2_O−α-pyr−CH_3_]^−^ | Psi-bufarenogin | c |
|  | C_24_H_32_O_6_ | 9.23 | 417.2275 | 0.9 | 417.2275[M+H]^+^  439.2096[M+Na]^+^  399.2173[M+H−H_2_O]^+^  371.2211[M+H−H_2_O−CO]^+^ | Bufotalidin | c |
|  | C_41_H_68_O_13_ | 10.13 | 783.4872 | −2.1 | 783.4872[M+H]^+^  441.3720[M+H−H_2_O−2Glc]^+^  423.3610[M+H−2H_2_O−2Glc]^+^ | Ginsenoside La | a |
|  | C_30_H_48_O_3_ | 10.69 | 457.3666 | −2.3 | 457.3666[M+H]^+^  439.3586[M+H−H_2_O]^+^  421.3483[M+H−2H_2_O]^+^ | 16-Oxoseratenediol | a |
|  | C_9_H_10_O_3_ | 11.03 | 167.0708 | 3.5 | 167.0708[M+H]^+^  93.0347[M+H−CH_3_CO−CH_3_O]^+^ | Paeonol | a |
|  | C_6_H_12_O_6_ | 12.06 | 534.1936 | −0.8 | 534.1936[M+HCOO]^−^  397.1625[M−H−C_2_H_5_NO_3_]^−^ | Galactose | a, h |
|  | C_24_H_32_O_6_ | 12.21 | 473.2178 | 1.7 | 473.2178[M+H]^+^  377.1715[M+H−α-pyr]^+^  359.1651[M+H−H_2_O−α-pyr]^+^ | Bufarenogin | c |
|  | C_26_H_45_NO_7_S | 12.88 | 516.2979 | −2 | 516.2979[M+H]^+^  480.2805[M+H−2H_2_O]^+^  462.2668[M+H−3H_2_O]^+^  337.2517[M+H−3H_2_O−Tau]^+^  126.0220[Tau]^+^ | Taurocholic acid | g |
|  | C_42_H_72_O_14_ | 12.97 | 845.4927 | 2.7 | 845.4927[M+HCOO]^−^  799.4867[M−H]^−^  783.4883[M−H−H_2_O]^−^  637.4105[M−H−Glc]^−^  161.0436[Glc]^−^ | Ginsenoside Rg_1_ | a |
|  | C_24_H_30_O_6_ | 13.11 | 415.212 | 1.2 | 457.3679[M+H]^+^  397.1994[M+H−H_2_O]^+^  301.2499[M+H−H_2_O−α-pyr]^+^ | 19-Oxodesacetylcinobufagin | c |
|  | C_30_H_48_O_3_ | 13.15 | 457.3679 | 0.6 | 457.3679[M+H]^+^  439.3573[M+H−H_2_O]^+^  421.3467[M+H−2H_2_O]^+^ | Oleanolic acid | a, b |
|  | C_42_H_72_O_14_ | 13.19 | 801.4996 | 0.1 | 823.4800[M+Na]^+^  801.4996[M+H]^+^  603.42470[M+H−2H_2_O−Glc]^+^  585.4120[M+H−3H_2_O−Glc]^+^ | Ginsenoside Rf | a, b |
|  | C_26_H_43_NO_6_ | 13.34 | 478.3153 | −4.4 | 478.3153[M+HCOO]^−^  327.2627[M−H−gly−2CH_3_]^−^  311.2639[M−H−gly−2CH_3_−O]^−^ | Glycocholic acid | e, g |
|  | C_24_H_32_O_5_ | 13.41 | 401.2326 | 1 | 401.2326[M+H]^+^  383.2170[M+H−H_2_O]^+^  365.2065[M+H−2H_2_O]^+^  347.1996[M+H−3H_2_O]^+^ | Resibufaginol | c |
|  | C_36_H_62_O_10_ | 13.50 | 655.4413 | −0.4 | 655.4413[M+H]^+^  603.4237[M+H−2H_2_O−O]^+^  585.4097[M+H−3H_2_O−O]^+^ | Pseudoginsenoside RT_5_ | a |
|  | C_26_H_42_NNaO_6_ | 13.83 | 488.298 | −0.6 | 488.2980[M+H]^+^  337.253[M+H−Na−3H_2_O−Gly]^+^ | Sodium glycocholate | e, g |
|  | C_48_H_76_O_19_ | 13.86 | 979.4856 | −1.7 | 979.4856[M+Na]^+^  603.4229[M+H−Glc−Glc-acid−H_2_O]^+^ | Ginsenoside Ro | a, b |
|  | C_48_H_82_O_19_ | 13.89 | 985.5356 | 1.4 | 985.5356[M+Na]^+^  766.4859[M+H−Glc−H_2_O−O]^+^ | 20-Glucoginsenoside Rf | a |
|  | C_54_H_92_O_23_ | 14.16 | 1107.5976 | 1.8 | 1107.5976[M−H]^−^  1153.6048[M+HCOO]^−^  945.5370[M−H−Glc]^−^  783.4867[M−H−2Glc]^−^  621.4408[M−H−3Glc]^−^ | *Pseudoginsenoside D | a |
|  | C_41_H_70_O_13_ | 14.2 | 771.4877 | −1.5 | 771.4877[M+H]^+^  605.4386[M+H−H_2_O−Xyl−O]^+^  441.3715[M+H−2H_2_O−Xyl−Glc]^+^  425.3778[M+H−2H_2_O−Xyl−Glc−O]^+^ | Notoginsenoside R_2_ | a |
|  | C_42_H_72_O_13_ | 14.23 | 785.5029 | −2.1 | 785.5029[M+H]^+^  605.4386[M+H−H_2_O−Glc]^+^  459.3815[M+H−2Glc]^+^  325.1133[2Glc]^+^ | Ginsenoside Rg_3_ | a, b |
|  | C_24_H_34_O_5_ | 14.44 | 403.2428 | 0.6 | 403.2428[M+H]^+^  349.2166[M+H−3H_2_O]^+^ | 1β-Hydroxybufalin | c |
|  | C_41_H_68_O_12_ | 14.5 | 753.4775 | −1.1 | 753.4775[M+H]^+^  573.4102[M+H−H_2_O−Glc]^+^ | Ginsenoside Rg_5_ | a |
|  | C_24_H_32_O_5_ | 14.83 | 401.2325 | 0.7 | 401.2325[M+H]^+^  365.2127[M+H−2H_2_O]^+^  347.2006[M+H−3H_2_O]^+^ | Desacetylcinobufagin | c |
|  | C_36_H_60_O_8_ | 14.94 | 621.4366 | 0.8 | 621.4366[M+H]^+^  603.4243[M+H−H_2_O]^+^  441.372[M+H−H_2_O−Glc]^+^ | Ginsenoside Rh_4_ | a |
|  | C_53_H_90_O_22_ | 14.95 | 1079.5986 | −1.0 | 1079.5986[M+H]^+^  1104.7550[M+Na]^+^  929.54[M+H−H_2_O−Fur] ^+^ | Ginsenoside Rc | a, b |
|  | C_26_H_36_O_6_ | 15.17 | 445.2588 | 0.6 | 445.2588[M+H] ^+^  467.2393[M+Na] ^+^  409.2319[M+H−2H_2_O]^+^  385.2365[M+H−H_2_O−CH_2_CO]^+^  367.2268[M+H−2H_2_O−CH_2_CO]^+^  349.2162[M+H−α-pyr]^+^  331.1999[M+H−H_2_O−α-pyr]^+^  271.2065[M+H−2H_2_O−CH_2_CO−α-pyr]^+^ | *Bufotalin | c |
|  | C_56_H_94_O_24_ | 15.21 | 1195.6054 | −4.4 | 1195.6054[M+HCOO]^−^  1149.6032[M−H]^−^  943.5289[M−H−CH_3_CO−Glc]^−^  781.4687[M−H−CH_3_CO−2Glc]^−^ | Quinquenoside R_1_ | a |
|  | C_42_H_72_O_13_ | 15.23 | 807.485 | −1.9 | 807.4850[M+Na]^+^  621.4353[M+H−H_2_O−Rha]^+^  423.36173[M+H−3H_2_O−Rha−Glc]^+^ | Ginsenoside Rg_2_ | a |
|  | C_36_H_54_O_10_ | 15.26 | 647.3804 | 2.1 | 647.3804[M+H]^+^  385.2364[M+H−GlcUA−C_6_H_12_]^+^  367.2268[M+H−H_2_O−GlcUA−C_6_H_12_]^+^ | Gypsogenin-3-glucoronide | a |
|  | C_26_H_32_O_7_ | 15.41 | 457.2223 | 0.5 | 457.2223[M+H]^+^  439.3511[M+H−H_2_O]^+^  332.1877[M+H−α-pyr−CHO]^+^ | 19-Oxocinobufagin | c |
|  | C_48_H_82_O_19_ | 15.64 | 991.5519 | 3.6 | 991.5519[M+HCOO]^−^  783.4906[M−H−Glc]^−^  621.4358[M−H−2Glc]^−^ | *Ginsenoside Rd | a, b |
|  | C_42_H_70_O_12_ | 15.68 | 767.4924 | −2.1 | 767.4924[M+H]^+^  605.4392[M+H−Rha−O]^+^  443.3875[M+H−Rha−Glc−O]^+^ | Ginsenoside Rg_4_ | a |
|  | C_18_H_32_O_16_ | 15.71 | 505.1781 | 3.5 | 505.1781[M+H]^+^  487.1651[M+H−H_2_O]^+^  325.1127[M+H−H_2_O−Glc]^+^  163.0503[M+H−H_2_O−2Glc]^+^ | Panose | a |
|  | C_36_H_62_O_9_ | 15.76 | 683.4398 | 3.2 | 683.4398[M+HCOO]^−^  637.4327[M−H]^−^  475.3783[M−H−Glc]^−^  391.2803[M−H−Glc−C_6_H_12_]^−^  161.0439[Glc]^−^ | Ginsenoside Rh_1_ | a |
|  | C_26_H_34_O_7_ | 15.79 | 459.2373 | −0.9 | 459.2373[M+H]^+^  381.2055[M+H−H_2_O−α-pyr]^+^  363.1952[M+H−α-pyr]^+^ | Cinobufaginol | c |
|  | C_14_H_24_O | 15.81 | 231.1718 | −0.8 | 231.1718[M+Na]^+^  213.1633[M+H−H_2_O]^+^  147.1169[M+H−H_2_O−C_3_H_7_]^+^ | 1-Ethynylcyclododecanol | f |
|  | C_36_H_62_O_9_ | 15.82 | 639.4436 | −4.8 | 661.4256[M+Na]^+^  639.4436[M+H]^+^  459.3836[M+H−Glc−H_2_O]^+^  423.3620[M+H−3H_2_O−Glc]^+^  405.3513[M+H−4H_2_0−Glc]^+^ | Ginsenoside F_1_ | a |
|  | C_15_H_24_O | 15.85 | 221.1896 | −1.6 | 221.1896[M+H]^+^  203.1793[M+H−H_2_O]^+^  187.1480[M−H_2_O−CH_3_]^+^ | Spathulenol | a, b |
|  | C_28_H_48_O | 15.91 | 423.3617 | 4.6 | 423.3617[M+Na]^+^  383.2178[M+H−H_2_O]^+^  311.2708[M−H_2_O−C_5_H_11_]^+^ | Campesterol | a |
|  | C_26_H_44_NNaO_6_S | 16.31 | 522.2867 | 1.5 | 522.2867[M+H]^+^  339.2702[M+H−Na−2H_2_O−Tau]^+^ | Taurohyodeoxycholic acid sodium salt | e |
|  | C_47_H_80_O_17_ | 17.06 | 917.5485 | 1.8 | 939.5303[M+Na]^+^  917.5485[M+H]^+^  719.4701[M+H−2H_2_O−Glc]^+^  587.4297[M+H−2H_2_O−Xyl−Glc]^+^ | Gypenoside ix | b |
|  | C_36_H_62_O_8_ | 17.11 | 645.4357 | 3.1 | 645.4357[M+Na]^+^  587.4297[M+H−2H_2_O]^+^  425.3789[M+H−2H_2_O−Glc]^+^  407.3684[M+H−3H_2_O−Glc]^+^ | 20(S)-Protopanaxadiol saponins | a |
|  | C_23_H_38_O_2_ | 17.39 | 345.2795 | −1.1 | 345.2795[M−H]^−^  205.1576[M−H−C_10_H_20_]^−^ | 5-Resorcinol | a |
|  | C_24_H_40_O_5_ | 17.4 | 407.2798 | −1.3 | 453.2850[M+HCOO]^−^  407.2798[M−H]^−^  389.2656[M−H−H_2_O]^−^  363.2880[M−H−COO]^−^ | *Cholic acid | e, g |
|  | C_24_H_34_O_4_ | 17.49 | 387.2544 | 3.7 | 409.2367[M+Na]^+^  387.2544[M+H]^+^  369.2441[M+H−H_2_O]^+^  351.2336[M+H−2H_2_O]^+^  291.2113[M+H−α-pyr]^+^  255.2109[M+H−2H_2_O−α-pyr]^+^ | *Bufalin | c |
|  | C_17_H_22_O_2_ | 17.66 | 259.169 | −1.0 | 259.1690[M+H]^+^  227.1803[M+H−2O]^+^  199.1488[M+H−2O−C_2_H_4_]^+^ | Ginsenoyne A | a |
|  | C_15_H_18_ | 17.68 | 199.1488 | 3.2 | 199.1488[M+H]^+^  184.1220[M+H-CH_3_]^+^  169.1018[M+H-2CH_3_]^+^ | Cadalene | b |
|  | C_24_H_39_NaO_5_ | 17.71 | 431.2765 | −0.6 | 431.2765[M+H]^+^  373.2751[M+H−Na−2H_2_O]^+^  355.2647[M+H−Na−3H_2_O]^+^  337.2539[M+H−Na−4H_2_O]^+^  254.2035[M+H−Na−3H_2_O−C_5_H_9_O_2_]^+^ | Sodium cholate | g |
|  | C_32_H_46_O_8_ | 17.91 | 557.3112 | −1.4 | 557.3112[M−H]^−^  461.283[M−H−α-pyr]^−^  383.2164[M−H−SA]^−^  173.0804[SA−H]^−^ | Gamabufotalin-3-hydrogen suberate | c |
|  | C_26_H_45_NO_6_S | 17.96 | 500.3028 | −2.5 | 500.3028[M+H]^+^  339.268[M+H−2H_2_O−Tau]^+^ | Tauroursodeoxycholic acid | e, g |
|  | C_26_H_45_NO_6_ | 18 | 450.3213 | −0.1 | 472.3027[M+Na]^+^  450.3213[M+H]^+^  339.2680[M+H−2H_2_O−Gly]^+^  297.2643[M+H−2H_2_O−Gly−CH_2_CO]^+^ | Glycodeoxycholic acid | e, g |
|  | C2_6_H_42_NNaO_5_ | 18.21 | 472.3027 | −1.3 | 472.3027[M+H]^+^  339.2683[M+H−Na−2H_2_O−Gly]^+^  297.2643[M+H−Na−2H_2_O−Gly−CH_2_CO]^+^ | Sodium glycodeoxycholate | e |
|  | C_36_H_60_O_7_ | 18.48 | 605.4411 | −0.2 | 621.4298[M+Na]^+^  605.4411[M+H]^+^  425.3780[M+H−H_2_O−Glc]^+^  407.3673[M+H−2H_2_O−Glc]^+^ | Ginsenoside Rh_3_ | a |
|  | C_10_H_16_O | 18.51 | 153.1277 | 2.3 | 153.1277[M+H]^+^  123.1168[M+H−2CH_3_]^+^ | Isopulegone | b |
|  | C_19_H_32_O_2_ | 18.58 | 315.2297 | 0.8 | 315.2297[M+Na]^+^  257.2265[M+H−2H_2_O]^+^ | Dihydroandrosterone | f |
|  | C_30_H_48_O_5_ | 18.61 | 489.3577 | 0.4 | 489.3577[M+H]^+^  471.3469[M+H−H_2_O]^+^  453.3401[M+H−2H_2_O]^+^  435.3232[M+H−3H_2_O]^+^ | Asiatic acid | d |
|  | C_38_H_58_N_4_O_8_ | 18.82 | 699.4322 | −0.7 | 699.4322[M+H]^+^  681.4213[M+H−H_2_O]^+^  351.2333[M+H−H_2_O−Sub]^+^ | Bufalitoxin | c |
|  | C_24_H_38_O_4_ | 18.94 | 389.2688 | −2.3 | 435.2749[M+HCOO]^−^  389.2688[M−H]^−^  374.2522[M−H−CH_3_]^−^  332.2039[M−H−C_4_H_9_]^−^ | Diisooctyl phthalate | a |
|  | C_26_H_34_O_6_ | 19.02 | 443.2428 | 0 | 465.2243[M+Na]^+^  443.2428[M+H]^+^  347.2005[M+H−α−pyr]^+^  425.2307[M+H−H_2_O]^+^  383.2217[M+H−H_2_O−CH_2_CO]^+^  365.2111[M+H−2H_2_O−CH_2_CO]^+^  337.2166[M+H−2H_2_O−CH_2_CO−CO]^+^  319.2060[M+H−3H_2_O−CH_2_CO−CO]^+^ | *Cinobufagin | c |
|  | C_24_H_30_O_5_ | 19.26 | 399.2164 | −0.5 | 399.2164[M+H]^+^  267.2266[M+H−2O]^+^  271.2057[M+H−α-pyr−2O]^+^ | 3-Hydroxy-19-oxo-14, 15-epoxybufa-20,22-dienolide | c |
|  | C_32_H_44_O_9_ | 19.28 | 595.2865 | −2.0 | 595.2865[M+Na]^+^  399.2169[M+H−SA]^+^  271.2057[M+H−SA−α-pyr−2O]^+^ | Arenobufagin 3-hemisuberate | c |
|  | C_24_H_32_O_4_ | 19.32 | 385.2372 | −0.3 | 407.2186[M+Na]^+^  385.2372[M+H]^+^  253.1949[M+H−2H_2_O−α-pyr]^+^ | *Resibufogenin | c |
|  | C_10_H_16_ | 19.34 | 137.1327 | 1.8 | 137.1327[M+H]^+^  121.1014[M−CH_3_]^+^  107.0861[M+H−2CH_3_]^+^ | Beta-pinene | b |
|  | C_30_H_50_O_2_ | 19.55 | 443.3869 | 3.2 | 443.3869[M+H]^+^  425.3776[M+H−H_2_O]^+^  407.3664[M+H−2H_2_O]^+^ | Erythrodiol | d |
|  | C_19_H_34_O_2_ | 19.62 | 317.2438 | −4 | 317.2438[M+Na]^+^  137.1325[M+H−C_9_H_18_O_2_]^+^ | Methyl linoleate | a |
|  | C_24_H_39_NaO_4_ | 20.67 | 415.2822 | 0.8 | 415.2822[M+H]^+^  339.2690[M+H−2H_2_O−Na−OH]^+^ | Sodium deoxycholate | g |
|  | C_19_H_30_O_2_ | 20.74 | 313.2139 | 0.2 | 313.2139[M+Na]^+^  275.2003[M−CH_3_]^+^  257.1884[M−H_2_O−CH_3_]^+^ | Etiocholanolone | f |
|  | C_38_H_56_O_4_ | 20.79 | 599.4081 | 1.7 | 599.40813[M+Na]^+^  81.2788[M+H−Ferulic-acid]^+^ | Campesteryl ferulate | a |
|  | C_24_H_40_O_4_ | 20.85 | 393.3006 | 1.7 | 393.3006[M+H]^+^  375.2897[M+H−H_2_O]^+^  357.2793[M+H−2H_2_O]^+^  275.2003[M+H−H_2_O−C_5_H_9_O−CH_3_]^+^ | Murocholic acid | e |
|  | C_24_H_40_O_4_ | 20.98 | 391.2849 | −1.2 | 391.2849[M−H]^−^  373.2694[M−H−H_2_O]^−^  355.2620[M−H−2H_2_O]^−^ | Deoxycholic acid | e, g |
|  | C_16_H_28_O | 21.02 | 237.2217 | 1.6 | 237.2217[M+H]^+^  81.0707[M+H−C_10_H_19_O]^+^ | (1S,15S)-bicyclohexadecan | f |
|  | C_15_H_24_ | 21.05 | 227.1776 | 2.4 | 227.1776[M+Na]^+^  161.1329[M+H−C_3_H_8_]^+^ | α-Muurolene | a, b |
|  | C_30_H_50_O_3_ | 21.06 | 459.3841 | 1.7 | 459.3841[M+H]^+^  441.3733[M+H−H_2_O]^+^  423.3628[M+H−2H_2_O]^+^  339.2693[M+H−H_2_O−C_6_H_12_]^+^ | Dryobalanone | d |
|  | C_15_H_22_ | 21.08 | 203.1794 | −0.2 | 203.1794[M+H]^+^  161.1329[M+H−C_3_H_6_]^+^  147.1174[M+H−C_4_H_8_]^+^ | α-Curcumene | a |
|  | C_30_H_52_O_4_ | 21.11 | 477.3951 | 2.7 | 477.3951[M+H]^+^  459.3841[M+H−H_2_O]^+^  441.3733[M+H−2H_2_O]^+^  423.3628[M+H−3H_2_O]^+^ | Protopanaxatriol | a, b |
|  | C_20_H_40_N_2_O_8_ | 21.15 | 435.2772 | −2.7 | 435.2772[M−H]^−^  389.2665[M−H−H_2_O−CO]^−^ | 6-O-Hexonic acid | a |
|  | C_53_H_92_O_7_ | 21.36 | 841.6895 | −2.4 | 841.6895[M+H]^+^  703.5716[M+H−3H_2_O−C_6_H_12_]^+^ | Sitoindoside Ⅱ | a |
|  | C_30_H_50_O_2_ | 22.20 | 443.3904 | 4.7 | 443.3904[M+H]^+^  425.3778[M+H−H_2_O]^+^ | Dipterocarpol | d |
|  | C_12_H_25_NO | 22.38 | 200.2004 | −2.5 | 200.2004[M+H]^+^  184.1692[M−CH_3_]^+^ | Dimethyldecanamide | a |
|  | C_15_H_26_O_2_ | 22.42 | 239.2 | −2.2 | 239.2002[M+H]^+^  221.2264[M+H−H_2_O]^+^ | 4,10-Aromadendranediol | a |
|  | C_18_H_30_O_2_ | 22.78 | 279.2325 | 2.3 | 279.2325[M+H] ^+^  261.2206[M+H−H_2_O]^+^  95.0863[M+H−H_2_O−CH_2_CO−C_9_H_16_]^+^ | Linolenic acid | a |
|  | C_20_H_38_O_2_ | 22.86 | 311.2948 | 1.1 | 311.2948[M+H]^+^  250.1584[M+H−H_2_O−CH2CO]^+^ | Eicosenoic acid | a |
|  | C_34_H_46_O_9_ | 22.90 | 599.3223 | 1.5 | 621.3052[M+Na]^+^  599.3223[M+H]^+^  425.2326[M+H−SA]^+^ | Cinobufagin-3-hydrogen suberate | c |
|  | C_42_H_82_NO_10_P | 23.01 | 790.5596 | −1.0 | 790.5596[M−H]^−^  480.3076[M−H−CO_2_−C_18_H_34_O]^−^ | Phosphatidylserine | a |
|  | C_24_H_50_NO_7_P | 23.2 | 496.34 | 0.5 | 496.3400[M+H]^+^  478.3293[M+H−H_2_O]^+^  313.2740[M+H−C_5_H_15_NO_4_P]^+^  184.0736[M+H−C_19_H_37_O_3_]^+^ | Iysolecithin | g |
|  | C_16_H_25_N | 24.08 | 232.2064 | 1.7 | 232.2064[M+H]^+^  215.1766[M−CH_3_]^+^ | Muscopyridine | f |
|  | C_41_H_84_N_2_O_6_P | 24.17 | 731.6041 | −2.8 | 731.6041[M+H]^+^  184.0732[M+H−C_36_H_69_NO_2_]^+^ | Sphingomyelin | g |
|  | C_30_H_52_O_4_ | 24.34 | 499.3753 | −1.0 | 499.3753[M+Na]^+^  399.2156[M+H−H_2_O−2CH_3_]^+^ | Panaxatriol | b |
|  | C_24_H_40_O_3_ | 24.37 | 375.2894 | −3.0 | 375.2894[M−H]^−^ | Lithocholic acid | e, g |
|  | C_16_H_30_O_2_ | 25.00 | 277.2143 | 1.7 | 277.2143[M+Na]^+^ | 13-Tetradecen acetate | a |
|  | C_20_H_32_O_2_ | 25.54 | 305.2467 | −2.7 | 305.2467[M+H]^+^  289.2157[M−CH_3_]^+^  243.2094[M+H−CH_3_−HCOO]^+^ | Arachidonate | a |
|  | C_18_H_32_O_2_ | 27.13 | 279.2318 | −4.0 | 279.2318[M−H]^−^  179.1058[M−H−C_7_H_16_]^−^  163.1109[M−H−C_6_H_13_−OCH_3_]^−^ | Methyl heptadecadienoate | b |

Glc: glucose; Rha: rhamnose; Xyl: xylose; Gly: glycine; GlcUA: glucuronic acid; α-pyr: α-pyrone; Sub: suberylarginine; SA: suberic acid; Tau: taurine.

a *Panax ginseng C. A. Meyer*.

b *Panax notoginseng (Burk.) F. H. Chen*.

c *Venenum Bufonis*.

d *Borneolum.*

e Artificial Calculus Bovis.

f Artificial Moschus.

g Ox Bile Powder.

h Pearl.

* Identified by comparison with reference standards (Figure S1).
