# Supplementary material for: The Integrated Study on the Chemical Profiling to Explore the Constituents and Mechanism of Traditional Chinese Medicine Preparation Huatuo Jiuxin Pills Based on UPLC-Q-TOF/MSE and Network Pharmacology
Source: Front Mol Biosci. 2022 Mar 31;9:818285. doi: 10.3389/fmolb.2022.818285 (PMC9008511; doi:10.3389/fmolb.2022.818285)
Supplement: Supplementary file 3 [file Image1.pdf]

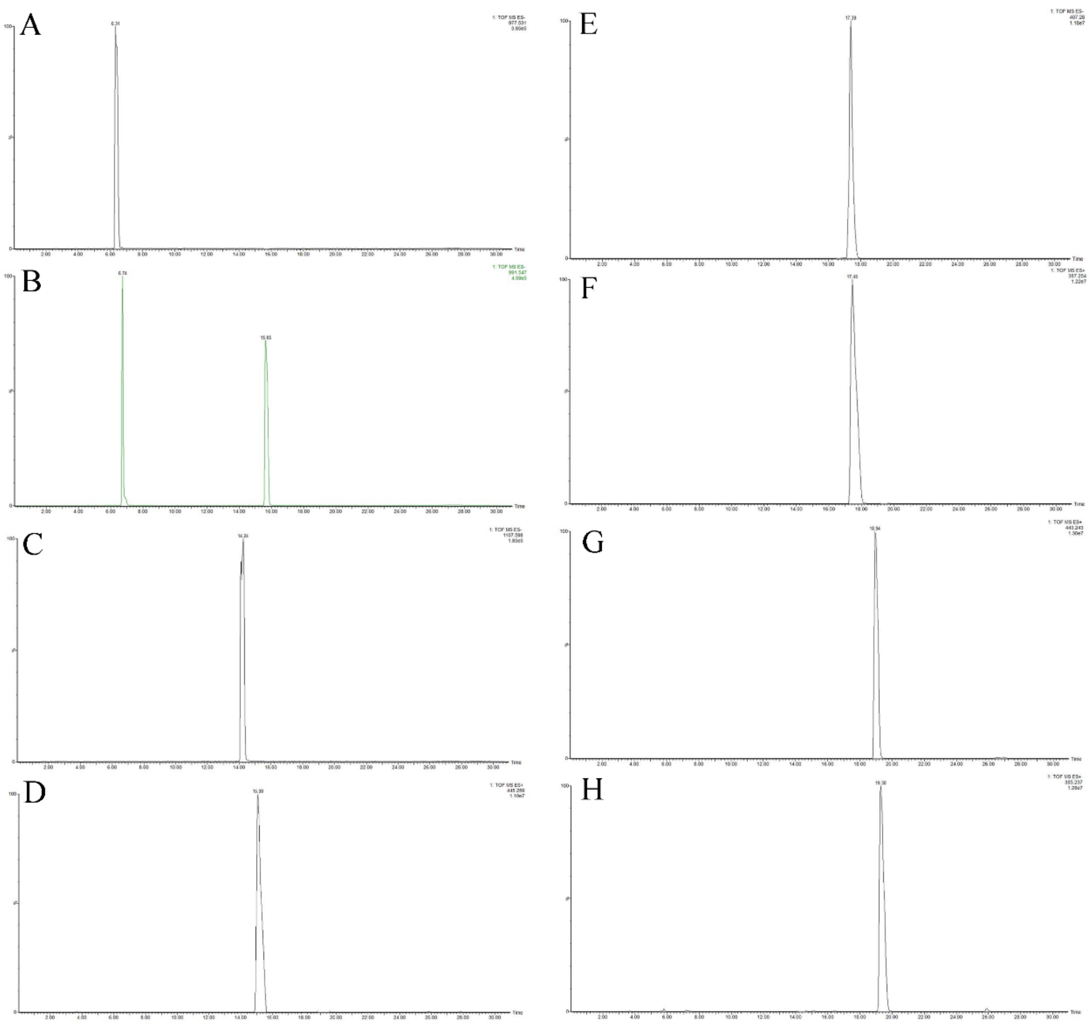

Fig. S1 The extracted ion chromatogram of reference

A. Notoginsenoside R1 B. Ginsenoside Re and Ginsenoside Rd C. Pseudoginsenoside D D. Bufotalin E. Cholic acid F. Bufalin G. Cinobufagin H. Resibufogenin
